# CRISPs Function to Boost Sperm Power Output and Motility

**DOI:** 10.3389/fcell.2021.693258

**Published:** 2021-08-05

**Authors:** Avinash S. Gaikwad, Ashwin Nandagiri, David L. Potter, Reza Nosrati, Anne E. O’Connor, Sameer Jadhav, Julio Soria, Ranganathan Prabhakar, Moira K. O’Bryan

**Affiliations:** ^1^School of Biological Sciences, Monash University, Clayton, VIC, Australia; ^2^School of BioSciences and Bio21 Institute, The Faculty of Science, The University of Melbourne, Parkville, VIC, Australia; ^3^Department of Chemical Engineering, Indian Institute of Technology Bombay, Mumbai, India; ^4^Department of Mechanical and Aerospace Engineering, Monash University, Clayton, VIC, Australia; ^5^Monash Micro Imaging – Advanced Optical Microscopy, Monash University, Clayton, VIC, Australia; ^6^Laboratory for Turbulence Research in Aerospace & Combustion (LTRAC), Department of Mechanical and Aerospace Engineering, Monash University, Clayton, VIC, Australia

**Keywords:** male fertility, male infertility, flagella, axoneme, crisp, sperm function

## Abstract

Fertilization requires sperm to travel long distances through the complex environment of the female reproductive tract. Despite the strong association between poor motility and infertility, the kinetics of sperm tail movement and the role individual proteins play in this process is poorly understood. Here, we use a high spatiotemporal sperm imaging system and an analysis protocol to define the role of CRISPs in the mechanobiology of sperm function. Each of CRISP1, CRISP2, and CRISP4 is required to optimize sperm flagellum waveform. Each plays an autonomous role in defining beat frequency, flexibility, and power dissipation. We thus posit that the expansion of the CRISP family from one member in basal vertebrates, to three in most mammals, and four in numerous rodents, represents an example of neofunctionalization wherein proteins with a common core function, boosting power output, have evolved to optimize different aspects of sperm tail performance.

## Introduction

Sperm motility is a critical determinant of male reproductive success *in vivo* (Mortimer, 1997; Suarez and Pacey, 2006). Sperm motility, or its absence, is a driver of evolution and is associated with male infertility in humans, agricultural and endangered species (Roldan et al., 2006). Despite this, flagellar waveform is rarely assessed in clinical or agricultural settings, but rather, tracking of the sperm head is used as a surrogate. Further, and despite great advances in our knowledge of the genes required to assemble a motile sperm tail (Pleuger et al., 2020; Touré et al., 2020), we know little of the processes required to activate and regulate sperm motility *in vivo*. Even in research settings, the effect of individual genes on sperm function is often inferred from the analysis of small numbers of sperm from tiny numbers of animals. As a consequence, such studies lack precision and are difficult to replicate. These knowledge gaps are due in large part to the challenges of imaging sperm flagellar motion and the absence of analytical processes to unravel the subtle changes in sperm flagellum function required to achieve optimal fertility. Recently, we have solved this challenge through the development of an imaging and analysis pipeline capable of mathematically quantifying the flagellar waveform with sufficient resolution to enable the measurement of kinematic characteristics such as the beat frequency and flagellar velocities and the spatiotemporal distribution of the hydrodynamic power dissipated along the tail (Nandagiri et al., 2021). Our approach takes advantage of the fact that many mammalian sperm are known to swim close to walls with their flagella beating in a plane that is parallel to the surfaces (Nosrati et al., 2015; Raveshi et al., 2021). We obtain high-resolution videos of sperm tethered at their heads to a glass slide. This tethered-cell assay enables us to collect data for large numbers (∼50) of beat cycles for a large number of sperm within a population. From such a dataset we can identify the average beat cycle in each population and systematically quantify differences induced by changes in genotype or media composition in a statistically meaningful manner.

We have now used this analysis pipeline to characterize the function of cysteine-rich secretory proteins (CRISPs) in sperm motility. CRISPs are a clade of the CRISP, Antigen 5, and Pathogenesis-related 1 (CAP) superfamily. They show a pronounced expression bias to the male reproductive tract in mammals. CRISPs contain a CAP domain, common to all superfamily members, and a CRISP domain composed of a hinge region and an ion channel regulatory region (ICR). The ICR domain has been implicated in ion channel regulation while the function of the CAP domain remains enigmatic (Gaikwad et al., 2020a). Sperm encounter CRISPs during all phases of development and maturation. CRISP2 is incorporated into the sperm acrosome, connecting piece and tail during spermatogenesis (O’Bryan et al., 2001); and in the mouse, sperm are surrounded by high concentrations of CRISP1 and its closely related paralogue CRISP4, during epididymal transit and sperm maturation (Nolan et al., 2006; Arévalo et al., 2020). None of the CRISPs are absolutely required for male fertility, although each of CRISP1, CRISP2, and CRISP4 are known to enhance sperm function, including motility (Da Ros et al., 2008; Gibbs et al., 2011; Turunen et al., 2012; Brukman et al., 2016; Hu et al., 2018; Lim et al., 2019). Exactly how this is achieved is unknown although based on biochemical data it is presumed that a significant proportion of activity will be mediated via ion channel, including at least some of which remain to be characterized (Gibbs et al., 2006, 2011; Ernesto et al., 2015). Deletion of all four CRISPs in mice leads to severe subfertility (Curci et al., 2020). For more information on the CRISPs and their roles in fertility and their evolutionary origins, readers are referred to (Gaikwad et al., 2020a).

Within this study we show that each CRISP acts independently to regulate flagellar waveform, power and, consequently, sperm velocity. CRISPs function to “boost” sperm power dissipation. We predict this will increase sperm competitiveness in polyandrous matings, thus describing a mechanism with which to underpin prior evolutionary data indicating CRISPs are the targets of positive Darwinian evolution.

## Materials and Methods

### Ethical Approval

All experimental procedures were carried out with approval of the Monash University Biological Sciences Animal Experimentation Ethics Committee and conducted in accordance with Australian National Health and Medical Research Council (NHMRC) Guidelines on Ethics in Animal Experimentation. *Crisp1*, *2*, *4*, and *Crisp1/4* double knockout mouse models were produced as previously described (Hu et al., 2018; Lim et al., 2019). Prior to use, genotype of all animals was confirmed by PCR analysis of tail biopsies as previously described (Hu et al., 2018; Lim et al., 2019).

### Sperm Sample Preparation

Sperm were collected from adult male mice (10–12 weeks old) using the back flushing technique and stored at 37°C in dark until used as described previously (Lim et al., 2019). Sperm were diluted to 1 × 10^5^ sperm/mL into modified Toyoda, Yokoyama and Hosi (TYH) media (135 mM NaCl, 4.8 mM KCl, 2 mM CaCl_2_, 1.2 mM KH_2_PO_4_, 1 mM MgSO_4_, 5.6 mM glucose, 0.5 mM Na-pyruvate, 10 mM L-lactate, and 10 mM HEPES, pH 7.4) containing 0.3 mg/ml BSA to facilitate the binding of sperm heads to the microscope slides (Toyoda et al., 1971; Lim et al., 2019). For imaging, a chamber was constructed on a glass slide with two parallel strips of double-sided tape of 90-micron nominal thickness spaced 16 mm apart, and 40 μl of the sperm suspensions was added and the chamber capped using a 17 mm^2^ No. 1.5 glass coverslip (Thermo Fisher Scientific). Head tethered sperm with free beating flagellum were imaged at room temperature (25°C) within 10–15 min of collection from the cauda epididymis at 400 fps for 10 s of which the first 1,000 frames were used in the analysis as described previously (Nandagiri et al., 2021). *N* = 25–30 sperm/genotype from at least 5–6 mice were imaged and analyzed.

### High-Speed Image Acquisition of Sperm and Image Analysis

The imaging system was constructed around an Olympus AX-70 upright microscope equipped with a U-DFA 18mm I.D. dark-field annulus, an UPlanAPO 20x 0.7 NA objective (Olympus, Japan) and incandescent illumination. In order to maximize system light efficiency, all extraneous optical elements were removed from the light paths. Images were captured on an ORCA-Flash4.0 v2+ sCMOS camera (Hamamatsu, Japan) streaming to a dedicated Firebird PCIe3 bus 1xCLD Camera Link frame grabber card (Active Silicon, United Kingdom). The image capture computer system utilized a Xeon E5-2667 12 core CPU running at 2.9 GHz with 64 Gb of DDR3 RAM and 1 TB of SSD hard drive space in a RAID0 configuration. Camera software control was affected by Fiji (ImageJ)^1^ (Schindelin et al., 2012) running the Multi-Dimensional Acquisition plugin under the Micro Manger Studio plugin v1.4.23^2^ (Edelstein et al., 2014). Acquisition was set to 4,000 time points with no time point interval and a 2 ms exposure time at 400 frames per second (fps) with the data saved as an image stack. The optical lateral resolution, at a 550 nm reference wavelength was 0.479 microns. The best-case optical lateral resolution, at Nyquist-Shannon sampling, was 0.650 microns (0.325 microns per pixel). Imaging at 512 × 512 pixels resulted in a sample field of view (FOV) of 166.4 × 166.4 microns. Mutant sperm which had a more rigid linear tail morphology necessitated an FOV increase, with a consequently minor reduction in the frame capture rate in order to capture the motion of the entire sperm. The best-case blur free motion capture of the system using 400 fps allowed element point velocities of 130 microns per second at Nyquist-Shannon frequency sampling to be measured. Mouse sperm are 110 μm in length and were divided into three parts: mid-piece (24 μm), principal piece (80 μm) and end piece (∼6 μm) for analysis (Nandagiri et al., 2021).

### Live-Dead Mouse Sperm Fluorescent Staining

Sperm viability over time was assessed using the LIVE/DEAD^TM^ Sperm viability kit as recommended by the manufacturer (Thermo Fisher Scientific).

### Proper Orthogonal Decomposition

Image-analysis protocols are used to extract smooth centerlines of sperm in every frame of the recorded image sequence (Nandagiri et al., 2021). The tangent angle at any point on the centerline is the angle made by the tangent at that point with the horizontal axis. Henceforth, *s* denotes the arc-length coordinate along the centerline and τ denotes time. The tangent-angle data is obtained at discrete locations, *s*_1_, *s*_2_, *…, s_*M*_*, and at times, τ_1_, τ_2_, …, τ_*N*_. The proper orthogonal decomposition of the tangent angle profile has been described previously by Werner et al. (2014) and Nandagiri et al. (2021). With this technique, the tangent angle data is resolved into a weighted sum of *time-independent* tangent angle profiles known as the shape modes:

$$\psi \left(s_{m},\tau_{n}\right)=\psi_{0}\left(s_{m}\right)+\sum_{k=1}^{M}B_{k}\left(\tau_{n}\right)\psi_{k}\left(s_{m}\right).$$

Here, ψ_0_ is the time average of the tangent angle observed at each *s_m_* and *B_k_* is the relative weight of the *k*-th shape mode, ψ_*k*_. The mean shape ψ_0_ and the shape modes ψ_*k*_ represent the entire set of shapes in the flagellar beating pattern. They have the units of radians. The dimensionless weight, *B*_*k*_, of the *k*-th shape mode is referred to as a shape coefficient.

The procedure to find the shape modes and the coefficients is summarized here. The values of the deviation *X*_*nm*_ of ψ from the mean ψ_0_, *X*_*nm*_ = ψ - ψ_0_, is an *N* × *M* matrix. In matrix form, we can therefore write X = B ⋅ ψ, where the dot signifies matrix multiplication, the *k* -th column of ψ corresponds to the M values of *k* -th shape mode vector, _*k*_, and the (*k*, *m*) -th element of B is *B*_*k*_ (τ_n_). The values of B and can be uniquely obtained by singular-value decomposition (SVD) of X which is calculated as follows (Ma et al., 2014; Werner et al., 2014). The *M* × *M* covariance matrix *C*_*X*_ = *X*^*T*^ ⋅ *X* was first calculated. Its eigenvectors arranged as columns gives ψ. The matrix *X* ⋅ Ψ^*T*^.

### Hydrodynamic Dissipation From Resistive Force Theory

From the smooth tangent angle profiles obtained by image-analysis and the proper orthogonal decomposition, we can calculate the instantaneous tangent and normal vectors, $\vec{t}$ and $\vec{n}$, and the velocity profile, $\vec{u}$, as functions of the local arc-length coordinate along the flagellar centerline, *s*, and the time, τ. The sperm flagellum operates in the low Reynolds number regime of fluid flow where the inertial forces are very small relative to the viscous forces. In this regime, the hydrodynamic dissipation can be calculated from the flagellar velocities using local Resistive Force Theory [RFT (Katz et al., 1975; Nandagiri et al., 2021)]. The hydrodynamic force per unit length at each point on the sperm body are directly related to the local velocities:

$$\vec{f}\left(s,\tau \right)=\zeta_{t}\vec{u_{t}}+\zeta_{n}\vec{u_{n}},$$

where, ζ_*t*_ and ζ_*n*_ are the local friction coefficients in the tangential and normal directions, and $\vec{u_{t}}$ and $\vec{u_{n}}$ are the instantaneous local tangential and normal velocities obtained from the image-analysis algorithm.

From RFT, local friction coefficients that consider the presence of the nearby surface are used with a linear taper in the flagellar diameter *a*:

$$\zeta_{t}=\frac{2\pi \mu }{\ln\frac{2h}{a}}$$

$$\zeta_{n}=\frac{4\pi \mu }{\ln\frac{2h}{a}}$$

where, the flagellar diameter *a* has a linear taper:

$$a=\left(a_{h}-a_{t}\right)\left(L-s\right)/L+a_{t},$$

with *a*_*h*_ = 0.57μm and *a*_*t*_ = 0.18μm as the radii at the head and tail end, respectively, and *L* = 110 μm. Since the head is tethered and the tail is nearly parallel to the surface, the distance of the tail from the surface *h* is approximated as *h* = *a*_*h*_, which is the radius at the head (Nandagiri et al., 2021).

The instantaneous contribution of the motion of each point of the sperm body to the power dissipated in the ambient fluid due to viscous friction is then:

$$p\left(s,\tau \right)=\vec{f}\left(s,\tau \right)\cdot \vec{u}\left(s,\tau \right).$$

We refer to this as the hydrodynamic dissipation. The time-average of the power dissipation per unit length at each location along the tail over several beat cycles is then given by:

$$\bar{p}\left(s\right)=\frac{1}{T}\int_{0}^{\text{T}}p\left(s,\tau \right)\text{d}\tau ,$$

where, *T* is the total time duration. The rate of hydrodynamic power dissipated by the whole cell is:

$$E=\int_{0}^{L}\bar{p}\left(s\right)\text{d}s.$$

The integrals are evaluated as discrete sums along the discrete centreline and the time points.

### Projected Free-Swimming Trajectories From Hydrodynamics

The hydrodynamic force distribution, $\vec{f}\left(s,\tau \right),$ was used to compute a hypothetical free-swimming trajectory for the sperm. This is the path that would be followed by the sperm if the tether was removed. In these calculations, it is assumed that the shape of the flagellar beat does not change after untethering.

We first assumed that the unknown free-swimming velocity is $\vec{U}$. The total hydrodynamic force on the hypothetical free-swimmer must be zero since the inertia is negligible. Each segment of the free-swimmer than moves at a velocity $\vec{u}+\vec{U}$ i.e., the sum of the undulatory velocity and the velocity of translation. Therefore, the net hydrodynamic force:

$$\int_{0}^{L}\vec{\vec{\zeta }}\left(s,\tau \right)\cdot \left(\vec{u}\left(s,\tau \right)+\vec{U}\left(\tau \right)\right)\text{ds}=\vec{0}$$

where, $\vec{\vec{\zeta }}=\zeta_{t}\vec{t}\vec{t}+\zeta_{n}\vec{n}\vec{n}$ is the hydrodynamic friction tensor. Re-arranging, we get the following expression for the unknown velocity of translation:

$$\vec{U}\left(\tau \right)=-\mu \left(\tau \right)\int_{0}^{L}\vec{\vec{\zeta }}\left(s,\tau \right).\vec{u}\left(s,\tau \right)\text{ds},$$

where,

$$\mu \left(\tau \right)={\left(\int_{0}^{L}\vec{\vec{\zeta }}\left(s,\tau \right)ds\right)}^{-1}.$$

The projected trajectory $\vec{R}\left(\tau \right)$ of the hypothetical free-swimming sperm can then be computed by integrating the velocity of translation $\vec{U}\left(\tau \right)$:

$$\vec{R}\left(\tau \right)=\int_{0}^{\tau }\vec{U}\left(\tau \right)d\tau$$

The projected straight-line velocity (VSL) is then calculated from the net displacement per beat cycle:

$$\text{VSL}=\frac{\text{Net}\text{displacement}\text{in}\text{cycle}}{\mathrm{Time}\mathrm{period}}$$

### Generation of Recombinant Mouse CRISP1 and CRISP4 Proteins and Sperm Motility Rescue Assay

Recombinant mouse CRISP4 protein was expressed and purified as described previously and the same batch was used in this study (Gaikwad et al., 2020b). The expression of recombinant mouse CRISP1 protein was carried out by transiently expressing a pcDNA^TM^ 5/FRT/TO construct containing *Crisp1* (ENSMUSG00000025431) cDNA in Expi293F^TM^ cells (Thermo Fisher Scientific) as described in Volpert et al. (2014). The transient transfection was carried out using cells at a density of 3 × 10^6^ cells/ml, with 1 mg/ml polyethylenimine (PEI) (Polysciences, Inc., United States) at a ratio of 1:3 (DNA:PEI) and incubated at 37°C, 5% CO_2_ in suspension culture for 5 days on an orbital shaker rotating at 110–140 rpm. After a further 72 h of incubation, cells were harvested by centrifuged at 1,000 rpm at 4°C for 10–15 min. The supernatant was retained for protein purification and cells pellets were discarded. The cell supernatant was dialyzed against 10mM HEPES buffer, pH 6.5, using 10kDa cut-off dialysis tubing overnight. Post-dialysis, supernatant proteins were separated using ion exchange chromatography using a carboxymethyl cellulose (CM) resin column (GE Healthcare Life Sciences). Bound protein was eluted from the CM resin in 50, 100, and 150 mM NaCl elution buffer (Supplementary Figure 1). To assess the effects of recombinant mouse CRISP1 and CRISP4 proteins on sperm motility, the proteins were buffer exchanged into standard TYH buffer using Amicon Ultra-15 centrifugal filter concentrators. Denatured recombinant CRISP1 and CRISP4 were obtained by heat-treating the purified proteins at 95°C for 10 min and used as negative controls for functional assays.

For functional assays, individual recombinant CRISP proteins at a final concentration of 1 μM were added to 1 × 10^5^ sperm/ml in TYH media as described above.

### Statistical Analyses

The degree of difference between a group of representative beat patterns was quantified by the following Procrustes measure:

$$d=\sqrt{\begin{array}{c} \sum_{i,j}{\left(x^{\left(1\right)}\left(s_{j},\tau_{i}\right)-x^{\left(2\right)}\left(s_{j},\tau_{i}\right)\right)}^{2}\\ +{\left(y^{\left(1\right)}\left(s_{j},\tau_{i}\right)-y^{\left(2\right)}\left(s_{j},\tau_{i}\right)\right)}^{2} \end{array}}$$

where, *x* and *y* are the positional coordinates of group (1) and (2) at a phase-time *τ_*i*_* and at a location corresponding to the arc-length variable *s*_*j*_ along the flagellar centreline. Procrustes measures were compared pair-wise across the different genotypes. Larger values of *d* imply larger differences between the flagellar waveforms.

Data were analyzed using Systat Version 13. The effect of gene deletion on aspects of sperm function were evaluated with ANOVA. Data for individual mice was averaged and values were compared between mice. Where significant differences were detected among groups, *post-hoc t*-tests were used to evaluate which treatment combinations drove the interactions and main effects. Significant differences were indicated with ^∗^*P* < 0.05, ^∗∗^*P* < 0.01, ^∗∗∗^*P* < 0.001, and ^****^P < 0.0001.

## Results

### Sperm Flagellar Beating Parameters Are Conserved Between Mouse Strains

To quantify the kinematics of sperm motility, we used the high-resolution high-speed dark-field imaging system described in Nandagiri et al. (2021). In brief, sperm were collected from the cauda epididymis and imaged within 10–15 min, based on experiments revealing that motility parameter were unchanged up to 60 min post-collection (Supplementary Figure 2). Sperm were tethered to glass microscope slides and head-tethered cells were imaged over 10.0 s at 400 fps. As illustrated in the Supplementary Videos 1–8, wild type sperm from each of the C57BL/6J × C57BL/6N (*Crisp1^–/–^* colony), C57BL/6N (*Crisp2^–/–^* and *Crisp4^–/––^* colonies), and C57BL/6J × C57BL/6N (*Crisp1/4^–/–^* colony) genetic backgrounds were visually comparable and executed traveling-wave beating patterns. 25–30 sperm from 5 to 6 mice per mouse strain were analyzed for the parameters described below. Raw image data (25–30 sperm/genotype from 5 to 6 mice/strain) used in this study can be found at^3^.

**FIGURE 1:**
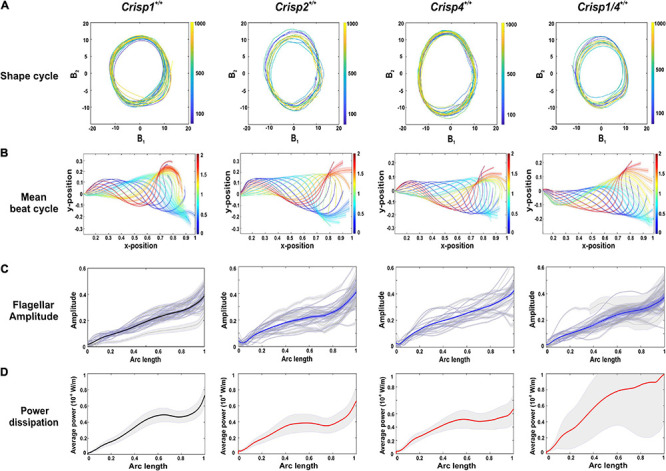
Analysis of flagellar oscillations and kinetics across sperm from wild type mouse strains. **(A)** A representative analysis of the shape cycle in B-space of flagellar betting over time as visualized by plotting coefficients (B_1_ and B_2_), the two most highly contributing POD modes (ψ_1_ and ψ_2_), against each other across the analyzed 1,000 frames. **(B)** A reconstruction of a representative beat cycle of sperm flagellum waveform from wild type mice from each strain. The error bars along the flagella indicates the ±SD in the sample. All lengths, including positional coordinates and the arc-length coordinate are normalized by the total sperm body length. The color spectrum represents the dimensionless fraction of the mean time-period. **(C)** Normalized spermatozoa flagellar beating amplitude from each mouse strain examined. Gray lines indicate the flagellar amplitude of individual sperm sample and blue lines indicate the mean flagellar amplitude. The gray zone along the flagella indicates the ± SEM in the sample. **(D)** An analysis of power dissipation (10^–8^ W/m) along the flagella. The bold line indicates averaged data across the sperm population ± SEM indicated in the gray zone. *Crisp1^+/+^* is denoted in black bold line as this will used as representative graph for comparison with knock out mice. *Crisp2^+/+^*, *Crisp4^+/+^*, and *Crisp1/4^+/+^* are denoted in bold red lines. For all of **(A–D)** the strain from which wild type mice and sperm were derived is indicated above each column and a total of *N* = 25–30 sperm/mouse strain were analyzed.

In order to precisely define the shape of the sperm flagella throughout the beating cycle, and over multiple cycles (the first 1,000 frames), centrelines were extracted from the image sequences and proper orthogonal decomposition (POD) (Werner et al., 2014) was used to resolve the two dimensional shape of each tail centreline, in each frame, into a weighted sum of distinct shape modes, The weighted combination of the first four of these modes ($\sum_{i=1}^{4}B_{i}\psi_{i}$) accounted for >98% of the total flagellum movement (Supplementary Figures 3A,B), with the weights (B_1_-B_4_) varying within a beat cycle and between cycles [i.e., each weight is a time-series B_*i*_(t)]. Among these four modes, Mode 1 (B_1_) and Mode 2 (B_2_) were dominant, accounting for 96% of the shapes over time. To a high degree of accuracy, therefore, every pair of *B*_1_ and *B*_2_ values, represents a unique tangent-angle profile. If the sequence of shapes is periodic in time, it will result in a cyclic trajectory when *B*_1_ is plotted *B*_2_. As shown by Nandagiri et al., such cycles can be used to systematically compare beating patterns of different sperm in a statistically meaningful manner. These coefficients were thus plotted against each other to aid comparison of beat cycles (Figure 1A). These data reveal that wild type sperm display repetitive and periodic flagellar oscillation that were indistinguishable between the four tested mouse strains.

To allow a quantitative assessment of variability between mouse strains, we used the extracted centreline data to construct a mean beating pattern for each sperm. Individual sperm waveform data, within a strain (Figure 1B), were then compared in terms of a Procrustes distance. The Procrustes distance quantifies the differences between waveforms and is defined as the root-mean-squared deviation between the flagellar shapes compared over a full representative beat cycle (intra-strain comparison, Supplementary Table 1). The beat cycles were then averaged to yield a representative beat pattern in the wild type population for each strain (Figure 1B), and a comparison of pairwise deviations made between each combination of wild type strain data to yield an inter-strain Procrustes distance (Table 1A). No significant differences in flagellar waveform were observed within or between wild type mouse strains (Supplementary Table 1).

**TABLE 1:** Comparative analysis of flagellar beating and kinematics across sperm from wild type and *Crisp* knock out mice.

**(A) The calculated Procrustes measure of flagellar beat across individual wild type mice lines. Wild type vs. Knock out**												
								**Procrustes measure ± SD**				***P* value**

*Crisp1^+/+^ vs. Crisp1^–/–^*								7.9 ± 1.8				<0.0001
*Crisp2^+/+^* vs. *Crisp2^–/–^*								4.5 ± 1.4				<0.001
*Crisp4^+/+^* vs. *Crisp4^–/–^*								7.6 ± 1.5				<0.0001
*Crisp1/4^+/+^* vs. *Crisp1/4^–/–^*								2.5 ± 1.1				<0.001

**(B) Analysis of power dissipation (in 10^–8^ J/s ±SD) at different regions of flagella in the presence or absence of *Crisp* genes**												

**Region on the flagella**	***Crisp1***		***P* Value**	***Crisp2***		***P* Value**	***Crisp4***		***P* Value**	***Crisp1/4***		***P* Value**
	**WT**	**KO**		**WT**	**KO**		**WT**	**KO**		**WT**	**KO**	

Mid-piece	1.5 ± 0.29	0.5 ± 0.15	<0.0001	1.5 ± 0.29	0.5 ± 0.3	<0.0001	1.6 ± 0.4	0.24 ± 0.09	<0.0001	1.5 ± 0.24	1.16 ± 0.39	<0.0001
Principal piece	4.03 ± 0.72	1.5 ± 0.34	<0.0001	4.4 ± 0.86	2.4 ± 0.8	<0.0001	4.1 ± 0.89	1.57 ± 0.35	<0.0001	4.1 ± 0.96	2.07 ± 0.46	<0.0001
End piece	5.4 ± 0.83	2.5 ± 0.6	<0.0001	5.3 ± 1.2	3.4 ± 0.72	<0.0001	5.1 ± 0.8	2.9 ± 0.76	<0.0001	5.2 ± 0.9	2.5 ± 0.69	<0.0001

*Please refer to Supplementary Table 4 for all the possible interactions between wild type vs. knock out genotype.*

**FIGURE 2:**
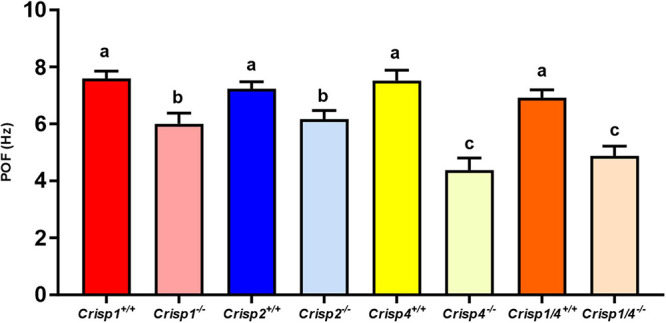
The calculated primary oscillating frequency (POF) for sperm from wild type and *Crisp* knock out mice. The data presented is 25–30 sperm/genotype. *N* = 5–6 mice/genotype. Different letters indicate significant differences between groups, where significance is measured relative to the strain-specific wild type. *P* < 0.01 for all marked “b”; and *P* < 0.001 for all marked “c” as determined using a two-way ANOVA. The intense color bars represent *Crisp* wild type mice data and lighter version of the same color bars represents *Crisp* knock out mice data, respectively (CRISP1 – red, CRISP2 – blue, CRISP4 – yellow, and CRISP1/4 – orange).

Subsequently, Fourier spectral analysis of the shape mode co-efficient (B_1_) and (B_2_) was undertaken to estimate the primary oscillating frequency (POF), a measure of the sperm tail beat frequency. The average POF wild type mouse sperm was 7.35 Hz and did not differ significantly between strains (Figure 2 and Supplementary Table 2).

**FIGURE 3:**
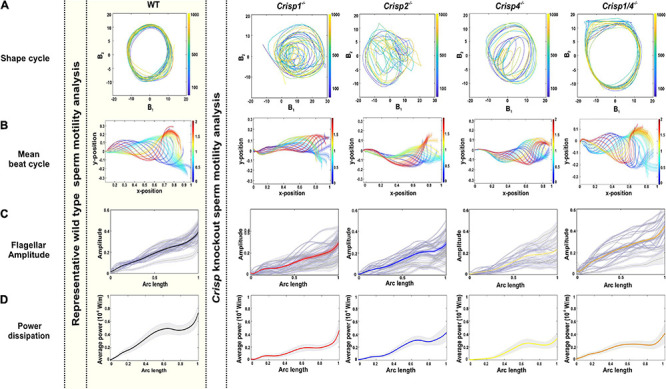
Effects of loss of CRISP1, CRISP2, CRISP4, and CRISP1/4 on sperm flagellar beating pattern. **(A)** The representative shape cycles of the flagellar beating across *Crisp* knockout mice. **(B)** Reconstruction of mean beat cycle of sperm across knock out mouse genotypes. *N* = 25–30 sperm/genotype were analyzed, and representative mean beat cycles are shown. The color spectrum represents the non-dimensional time scale. **(C)** Analysis of flagellar beating amplitude from sperm from individual knock out mouse genotypes. Gray lines indicate the flagellar amplitude of individual sperm and the respective bold lines indicate the mean flagellar amplitude. *Crisp1^–/–^* data is red, *Crisp2^–/–^* data is blue, *Crisp4^–/–^* data is yellow and *Crisp1/4^–/–^* data is orange **(D)** Analysis of power dissipation (10^–8^ W/m) along the flagella. The bold line indicates averaged data across the sperm population ± SEM indicated in gray zone. Mouse genotype is indicated above each column. For simplicity, *Crisp1* wild type data is shown as representative data for all wild types (within the shaded yellow box). *Crisp1^–/–^* data is red, *Crisp2^–/–^* data is blue, *Crisp4^–/–^* data is yellow and *Crisp1/4^–/–^* data is orange.

To evaluate swimming efficiency, the hydrodynamic power dissipation was calculated from the representative beat cycle of individual sperm using resistive force theory (Nandagiri et al., 2021). With regards to sperm, power dissipation is a measure of average power dissipated into the surrounding fluid at each point on the sperm flagellum. These data were comparable between sperm from the same strain, and between strains, and revealed that the power dissipation progressively increased along the tail (Figure 1D). We calculate that wild type sperm in a low viscosity medium (1 cP) dissipate 151 ± 5.05 fW across a beat cycle (Figure 3A). To complement these analyses, we measured the amplitude of the beating along the tail, including at set points corresponding to the mid-piece (24 μm), principal piece (80 μm), and end piece (∼6 μm) (Figure 1C). Flagellar amplitude was statistically comparable within and between mouse strains (Supplementary Tables 3–3.1).

In summary, sperm flagella waveform was highly repetitive, and predictable, between sperm within and between the mouse strains tested here. Regardless, for the analyses described below, sperm from each genetically modified mouse strain were compared to data from wild type littermates. For simplicity, *Crisp1* strain wild type data is shown in Figures 3A–D. All data can be found in Supplementary Figures.

### CRISPs Regulate Distinct Aspects of Sperm Flagellum Motility

**FIGURE 4:**
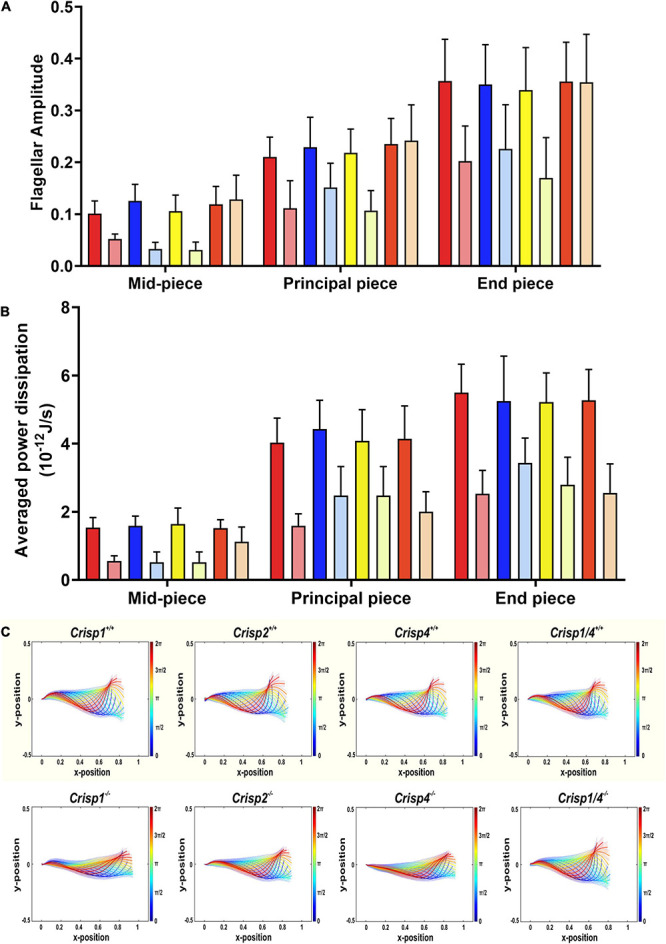
Qualitative and quantitative analysis of the population-averaged flagellar energetics and beat pattern across sperm from *Crisp* wild type and knock out mice. **(A)** Analysis of flagellar amplitude along the different regions of flagella starting in the mid-piece (24 μm), principal piece (80 μm) and end piece (∼6 μm). Differences were analyzed using a two-way ANOVA (±SD) on the mean sperm value from individual mice across genotypes. Please refer to Supplementary Tables 3, 3.1 for all the possible interactions between wild type versus knock out genotype. The *p* values indicate a statistically significant difference between wild type and respective null sperm. The intense color bars represent *Crisp* wild type mice and lighter version of the same color bars represents *Crisp* knock out mice, respectively (CRISP1 – red, CRISP2 – blue, CRISP4 – yellow, and CRISP1/4 – orange). **(B)** Analysis of power dissipation (10^–12^ J/s) per beat cycle along the different regions of the flagella – mid-piece, principal piece and end piece across wild type and knock out mice. Data presented is from a total number of 25–30 sperm/genotype. *N* = 5–6 mice/genotype. Data was analyzed by two-way ANOVA (±SD) on the mean sperm value from individual mouse genotypes. Comparisons were between sperm from wild type and knockout mice of each mouse strain, rather than between strains. The intense color bars represent *Crisp* wild type mice data and lighter version of the same color bars represents *Crisp* knock out mice data, respectively (CRISP1 – red, CRISP2 – blue, CRISP4 – yellow, and CRISP1/4 – orange). See Supplementary Table 4 for all the possible interactions between wild type versus knock out genotype. **(C)** Reconstruction of genotypic mean beat cycle of the sperm across four *Crisp* wild type (top panel) and *Crisp* knock out genotypes. The mean beat cycle of individual genotypes was constructed by analyzing 25–30 sperm/genotype. The color spectrum represents the non-dimensional time scale.

**FIGURE 5:**
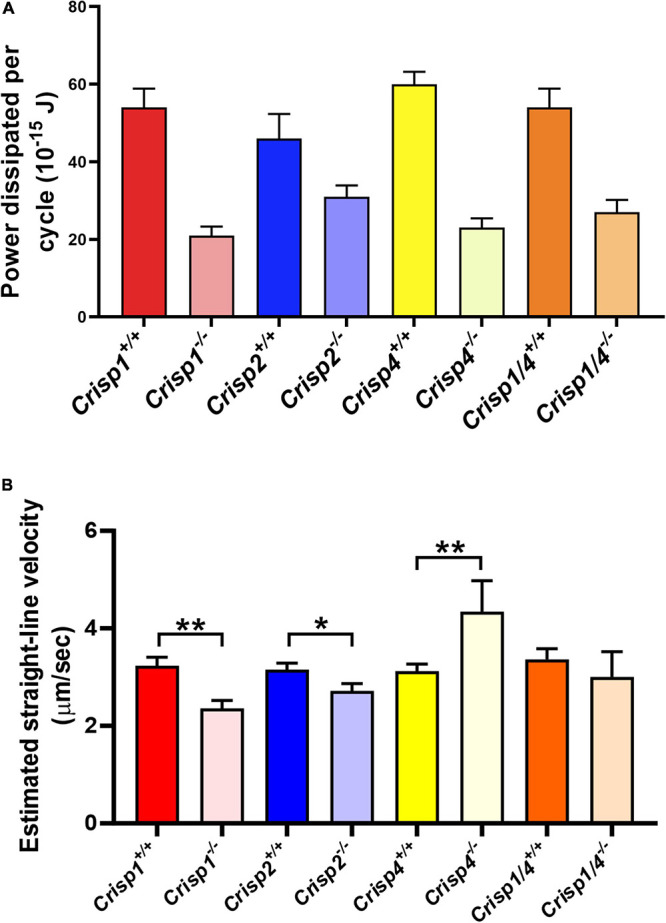
Mathematical estimation of flagellar kinetics of sperm across multiple genotypes. **(A)** The average hydrodynamic power dissipation (10^–15^ J) rate from spermatozoa from individual mouse strains as a function of genotype. The data presented is from a total number of 25–30 sperm/genotype. *N* = 5–6 mice/genotype. Data analyzed by two-way ANOVA (±SD) on the mean sperm value from individual mice across genotypes. The intense color bars represent *Crisp* wild type mice data and lighter version of the same color bars represents *Crisp* knock out mice data, respectively (CRISP1 – red, CRISP2 – blue, CRISP4 – yellow, and CRISP1/4 – orange). For all the possible interactions between wild type vs. knock out genotype please refer to Supplementary Tables 5, 5.1. **(B)** The calculated net progressive velocity (μm/sec) of sperm plus or minus the presence of individual CRISP genes. Data presented is from a total of 25–30 sperm/genotype. *N* = 5–6 mice/genotype, *P < 0.05, **P < 0.01, ***P < 0.001, and ****P < 0.0001 as analyzed by unpaired Student *t* test. Data presented as mean ± SD. The intense color bars represent *Crisp* wild type mice data and lighter version of the same color bars represents *Crisp* knock out mice data, respectively (CRISP1 – red, CRISP2 – blue, CRISP4 – yellow, and CRISP1/4 – orange).

In order to test the hypothesis that individual CRISPs play distinct roles in regulating sperm motility, sperm from each of *Crisp1*, *Crisp2*, *Crisp4*, and *Crisp1*/*4* double knockout, and wild type littermates were analyzed using the pipeline described above. This was initially done for the intracellular CRISP, CRISP2. As shown in Figure 2, the loss of CRISP2 reduced the POF of spermatozoa by 20% (*Crisp2^+/+^* 7.4 Hz vs. *Crisp2^–/–^* 5.9 Hz, *P* < 0.05, Supplementary Table 2). The shape cycle for sperm from *Crisp2^–/–^* mice had significant variability in the flagellar shapes between beat cycles (Figure 3A) (Procrustes distance *Crisp2^+/+^* c.f. *Crisp2^–/–^* mice, 4.5 ± 1.43, *P* < 0.001, Table 1A). The horizontal axis on the reconstructed mean beat cycle graph (Figure 3B), which corresponds to the length along the flagella, was used to determine the extent of restrained mobility in the flagella. Consistent with our prior work, the reconstructed mean beat cycle revealed that loss of CRISP2 lead to sperm with a stiff mid-piece (Figures 3B,C) (Lim et al., 2019), notably the first 36 μm (0.3 x-position on the X-axis) of the flagellum (Figure 3B). Sperm from *Crisp2^–/–^* mice also displayed a significantly lower flagellar amplitude along all regions of the tail compared to wild type counterparts (Figures 3C, 4A–C and Supplementary Table 3) and had a lower hydrodynamic power dissipation along the entire length of the flagellum (Table 1B, Figure 4B, and Supplementary Table 4). Overall, the loss of CRISP2 resulted in a 55% reduction in sperm power dissipation compared to wild type (*Crisp2^+/+^* 151 fW vs. *Crisp2^–/–^* 68 fW; *P* < 0.0001, Figure 5A and Supplementary Table 5).

Collectively, these data as reflected in the primary imaging data, reveal that CRISP2 plays a role in establishing flexibility principally in the sperm mid-piece, and in doing so, allows for an increase in flagella POF and amplitude. Nandagiri et al. (2021) have shown that the power dissipated hydrodynamically in the ambient fluid is proportional to the mechanical power delivered by the dynein motors in the axoneme. Therefore, the data effectively shows that the presence of CRISP2 boosts dynein power input.

The loss of the epididymal CRISP, CRISP1, also resulted in compromised flagellar function and was distinctly different to that seen in *Crisp2* null sperm. *Crisp1* deletion resulted in a significantly reduced POF compared to sperm from wild type mice (*Crisp1^+/+^* 7.3 Hz vs. *Crisp1^–/–^* 5.8 Hz, *P* < 0.05, Figure 2). The corresponding shape cycle revealed significant variability in the flagellar waveforms between beat cycles (Figure 3A), and thus a relatively large Procrustes distance between genotypes (7.9 ± 1.8, Table 1A). The mean beat cycle revealed that sperm from *Crisp1^–/–^* mice displayed a highly restrained flagella amplitude, wherein the region of “stiffness” extended through the mid-piece and into the principal piece up to a distance of 60 μm (Figures 3B, 4B and Supplementary Table 3). The loss of CRISP1 resulted in a 50 and 64% reduction in power dissipation from the mid-piece and principal piece of the flagella, respectively, when compared to wild type sperm (Table 1B and Figures 3D, 4B), and collectively a 65% reduction in power dissipation per cycle (*Crisp1^+/+^* 147 fW compared to *Crisp1^–/–^* 51 fW; *P* < 0.0001, Figure 5A and Supplementary Table 5). These data reveal that CRISP1 plays a role in defining the energetics of the mid-piece and proximal principal piece of the sperm tail and suggests that the “receptors” mediating CRISP1 function are located along the proximal region of the tail plasma membrane.

Sperm from *Crisp4^–/–^* mice also had a significantly decreased POF compared to controls (*Crisp4^+/+^* 7.5 Hz vs. *Crisp4^–/–^*4.1 Hz, *P* < 0.0001, Figure 2 and Supplementary Table 2), and their flagellar waveform were distinctly different to sperm from *Crisp1^–/–^* or *Crisp2^–/–^* mice. The shape cycle displayed irregular circular loops (Figure 3A) and the reconstructed mean beat cycle, as reflected in the original videos, revealed that sperm from *Crisp4^–/–^* mice were restrained along the entire length of the flagella compared to sperm from wild type littermates (Figure 3B). Of the sperm genotypes analyzed in this study, *Crisp4^–/–^* mouse sperm had the highest variation in flagellar kinetics (Procrustes distance value of 7.69 ± 1.5 compared to wild type littermates, Supplementary Table 1). The power dissipation for sperm from the *Crisp4^–/–^* was 86, 56, and 53% lower along the mid-piece, principal piece and end piece of the flagella, respectively, compared to sperm from wild type littermates (Table 1B and Figure 4B). Overall, the loss of CRISP4 resulted in a 65.5% reduction in power dissipated per sperm per cycle (*Crisp4^+/+^* 154 fW vs. *Crisp4^–/–^* 53 fW, *P* < 0.0001, Figure 5A and Supplementary Table 5). These data suggest that the molecules that mediate CRISP4 function on the sperm plasma membrane are distributed along the entire tail and that they ultimately feed into the energy generating machinery of the axoneme.

Our previous study showed that while sperm from *Crisp1^–/–^* mice had a compromised ability for progressive motility (Hu et al., 2018), somewhat surprisingly, sperm from *Crisp4^–/–^* mice displayed a significantly increased progressive motility compared to those from wild type littermates in aqueous media (Hu et al., 2018). To address this apparent conundrum, using the high-resolution data obtained in this study, we calculated the net displacement of individual sperm from each of the four genetically modified mouse lines by mathematically modeling individual sperm movement under untethered, free swimming conditions. The estimated progressive motility of sperm from *Crisp4^–/–^* mice in a low viscosity media was 36% higher than that of sperm from control mice (*Crisp4^+/+^* 3.3 μm/s vs. *Crisp4^–/–^* 4.5 μm/s, *P* < 0.01, Figure 5B and Supplementary Table 6) and thus qualitatively consistent with the computer assisted sperm analyser (CASA) measurements. Also, in accordance with the CASA data, the loss of CRISP1 or CRISP2 resulted in a reduced projected net progressive motility compared to control: by 33% for CRISP1 (*Crisp1^+/+^* 3.3 μm/s vs. *Crisp1^–/–^* 2.2 μm/s, *P* < 0.01) and 13% for CRISP2 (*Crisp2^+/+^* 3.1 μm/s compared to *Crisp2^–/–^* 2.7 μm/s, *P* < 0.05) (Figure 5B and Supplementary Table 6).

Depending on the species, sperm encounter CRISP1, and/or CRISP4 during epididymal maturation. The removal of all epididymal CRISPs from the mouse leads to compromised sperm function and fertilization potential (Hu et al., 2018), and as we have hypothesized previously, is likely to mirror the roles of the single CRISP expressed in the human epididymis (Hu et al., 2018). As revealed here, the beating pattern of sperm from *Crisp1/4* double knockout mice were superficially similar to sperm from wild type controls. There was a low Procrustes distance value between genotypes (2.5 ± 1.1, Table 1A) and flagellar amplitudes were comparable (Supplementary Tables 3–3.1). The combined deletion of *Crisp1* and *Crisp4* did not, however, rescue sperm functional competence. Sperm from *Crisp1/4^–/–^* mice had significantly reduced POF (*Crisp1/4^+/+^* 7.2 Hz vs. *Crisp1/4^–/–^* 4.8 Hz, *P* < 0.0001, Figure 3C and Supplementary Table 2) and a 59% reduction in power dissipation along the flagellum compared to wild type controls (*Crisp1/4^+/+^* 147 fW compared to *Crisp1/4^–/–^* 60 fW, *P* < 0.0001, Figure 5A and Supplementary Table 5). The loss of epididymis CRISPs also resulted in reduced estimated velocity (*Crisp1/4^+/+^* 3.5 μm/s vs. *Crisp1/4^–/–^* 2.9 μm/s, *P* < 0.05, Figure 5B and Supplementary Table 6) consistent with CASA data (Hu et al., 2018).

Collectively, these data reveal that in the mouse, CRISP1 and CRISP4 function to define different, potentially opposing, aspects of sperm flagella waveform, but collectively function to amplify the axonemal power generation. Further, and as a consequence of the observation that sperm from *Crisp1/4^–/–^* appeared to lose motility more quickly than sperm from wild type of single deletion mice, we undertook a temporal analysis of sperm motility. Paired LIVE/DEAD staining and motility analysis revealed that this was not due to elevated rates of death (Supplementary Figure 4), but rather sperm from *Crisp1/4^–/–^* mice rapidly became immotile after removal from the cauda epididymis.

The removal of any form of CRISP compromised flagellar waveform, power dissipation and POF (Figures 2–4). While the combined removal of CRISP1 and CRISP4 returned sperm flagellum shape to close to that seen in sperm from wild type mice, it did not rescue power. Each of the three CRISPs play a role in maximizing power output and each act autonomously (Figure 5A). Each of CRISP1, CRISP2 and CRISP4 have roles in defining POF, however, CRISP4 appears to have the dominant role and, as suggested by the non-statistically significant difference between *Crisp4* and *Crisp1/4* knock out mouse values, it appears to function up-stream of CRISP1 in the mouse (Figure 2).

### Recombinant Epididymal Mouse CRISPs Can Qualitatively and Quantitatively Rescue Sperm Motility Defects in Mouse

**FIGURE 6:**
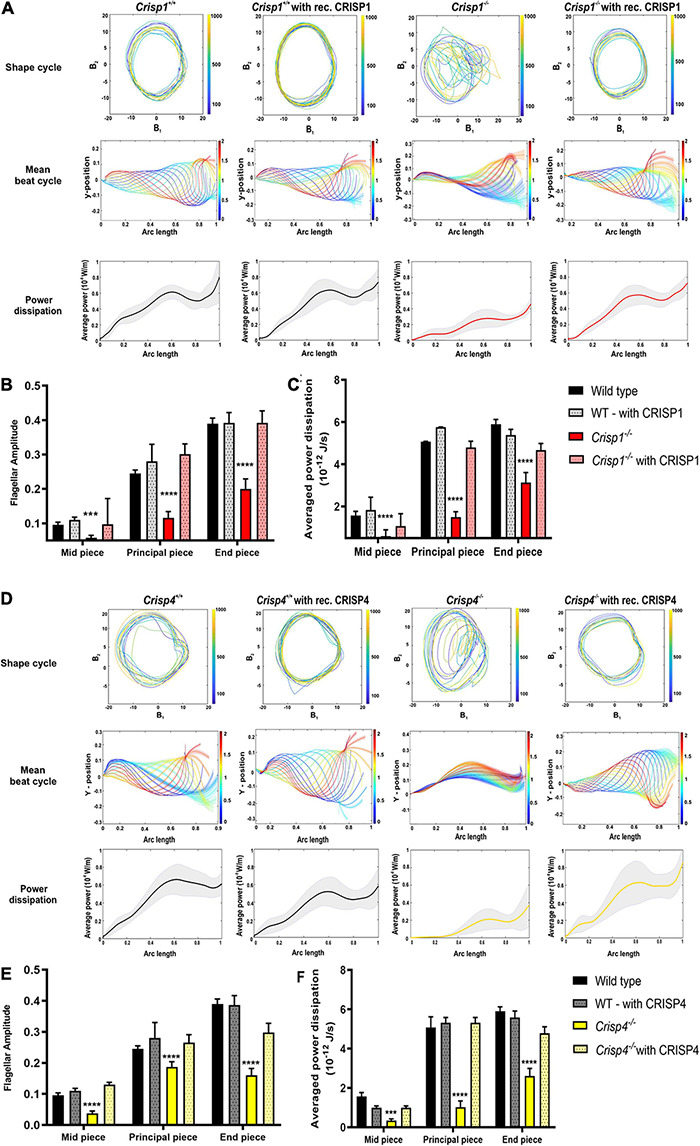
Effects of recombinant epididymal CRISPs on sperm from *Crisp1* and *Crisp4* wild type and knock out mice. **(A)** The representative shape cycles of sperm flagellar beating, reconstruction of mean beat cycle and analysis of power dissipation (10^–8^ W/m) along the flagella of the sperm from *Crisp1* wild type (black) and knock out (red) mice before (solid bar) and after (dotted bar) exposure to recombinant CRISP1 protein. *N* = 10–15 sperm/genotype were analyzed, and representative mean beat cycle and shape cycles of the flagellar beating is shown. For power dissipation, the black bold line indicates averaged data across the analyzed sperm population ± SEM. **(B)** Analysis of flagellar amplitude along the different regions of flagella (mid-piece, principal piece, and end piece) in sperm from *Crisp1* wild type and knock out mice before and after exposure to 1 μM recombinant CRISP1 protein. See Supplementary Table 7.1 for all the possible interactions between exposed and non-exposed genotype. **(C)** Analysis of power dissipation (10^–12^ J/s) along the different regions of flagella (mid-piece, principal piece, and end piece) in sperm from *Crisp1* wild type and knock out mice before and after exposure to 1 μM recombinant CRISP1 protein. The data presented is from a total number of 10–15 sperm/genotype. *** indicates *P* < 0.001 as analyzed by two-way ANOVA ± SEM. **** indicates *P* < 0.0001 as analyzed by two-way ANOVA ± SEM. See Supplementary Table 7.2 for all the possible interactions between exposed and non-exposed genotype. **(D)** The representative shape cycles of the flagellar beating, reconstruction of mean beat cycle and analysis of power dissipation (10^–8^ W/m) along the flagella of the sperm from *Crisp4* wild type (black) and knock out (yellow) mice before (solid bar) and after (dotted bar) exposure to 1 μM recombinant CRISP4 protein. *N* = 10–15 sperm/genotype were analyzed, and representative mean beat cycle and shape cycles of the flagellar beating is shown. For power dissipation, the black bold line indicates averaged data across the analyzed sperm population ± SEM. **(E)** Analysis of flagellar amplitude along the different regions of flagella (mid-piece, principal piece, and end piece) in sperm from *Crisp4* wild type and knock out mice before and after exposure to 1 μM recombinant CRISP4 protein. See Supplementary Table 8.1 for all the possible interactions between exposed and non-exposed genotype. **(F)** Analysis of power dissipation (10^–12^ J/s) along the different regions of flagella (mid-piece, principal piece, and end piece) in sperm from *Crisp4* wild type and knock out mice before and after exposure to1 μM recombinant CRISP4 protein. The data presented is from a total number of 10–15 sperm/genotype. *** indicates *P* < 0.001 as analyzed by two-way ANOVA ± SEM. **** indicates *P* < 0.0001 as analyzed by two-way ANOVA ± SEM. See Supplementary Table 8.2 for all the possible interactions between exposed and non-exposed genotype. All representative videos can be access via doi: https://doi.org/10.26180/5dbf4bff1acce.

To test if the phenotypic deficits induced by the loss of epididymal CRISPs can be rescued by *in vitro* exposure to the corresponding protein we produced recombinant CRISP1 and CRISP4 and exposed sperm to previously published physiologically relevant concentration of CRISPs *in vitro* (Ernesto et al., 2015). As illustrated in Supplementary Videos 9, 10 the addition of 1 μM of either recombinant CRISP1 or CRISP4 to sperm from wild type mice had no discernible effects on motility parameters (Figures 6A–F). By contrast, the motility defects seen in sperm from *Crisp1^–/–^* and *Crisp4^–/–^* mice were qualitatively and quantitatively rescued by exposure to 1 μM recombinant mouse CRISP1 (Supplementary Video 11) and CRISP4 (Supplementary Video 12), respectively (Figure 6). Raw image data used in this study can be found at^4^.

Specifically, incubation of sperm from *Crisp1^–/–^* and *Crisp4^–/–^* with the respective recombinant protein recovered the POF, flagellar amplitude, and power dissipation resulting in a mean beat cycle comparable to the sperm from wild type mice (Figure 6, Supplementary Figure 6A, and Supplementary Tables 7, 8). To test for functional redundancy between recombinant CRISPs, we further tested the effects of recombinant CRISP1 on sperm from *Crisp4^–/–^* mice and *vice versa*. No recovery of motility parameters was observed indicating that mouse CRISP1 and CRISP4 function non-redundantly in regulating *ex vivo* sperm motility (Supplementary Videos 1, 2 and Supplementary Figures 5B, 7).

By contrast, sperm from *Crisp1/4^–/–^* mice when exposed to individual recombinant CRISP1, CRISP4 or combined CRISP1 and 4, did not rescue the deficit in power dissipation (Supplementary Figures 6C, 8 and Supplementary Tables 10.1, 10.2), noting the waveform of sperm from wild type and *Crisp1/4^–/–^* was already comparable (as shown in Figure 3 and Supplementary Table 10.1). These data suggest CRISP1 and 4 play a temporally restrained role for epididymal CRISPs in epididymal sperm maturation and that this role can be mediated by either CRISP1 or CRISP4 i.e., they act redundantly.

## Discussion

Cysteine-rich secretory proteins are a sub-clade of the CAP protein superfamily. They are highly expressed in the male reproductive tract and incorporated into, and onto, sperm during every phase of their lifespan. CRISPs are not essential for fertility (Cohen et al., 2008; Hu et al., 2018; Lim et al., 2019), but as demonstrated here, they enable a stable and powerful form of sperm locomotion. We reveal, for the first time, that each of the three CRISPs that sperm encounter during spermatogenesis and epididymal maturation, regulate sperm tail flexibility, and POF to produce a waveform capable of optimal power dissipation and progressive motility. As reflected in evolutionary analyses (Gaikwad et al., 2020a), the acquisition and expansion of CRISP family member expression in the male reproductive tract represents a means to optimize sperm flagellar waveform and turbocharge sperm function, and thus likely success in situations of sperm competition.

The analysis of sperm from *Crisp1*, *Crisp2*, *Crisp4*, and *Crisp1/4* deficient mouse lines shows that CRISPs control multiple aspects of sperm motility, and that each CRISP contributes non-redundantly to optimize flagella waveform. CRISP2, which is incorporated internally into the sperm connecting piece and outer dense fibers (O’Bryan et al., 1998), regulates the flexibility of the mid-piece. CRISP1, which sperm encounter during epididymal maturation and data suggests signals via a plasma membrane localized receptor(s), regulates the flexibility of the proximal 60% of the flagellum. Mouse sperm are also bathed in CRISP4 during epididymal maturation, where it appears to play a superficially opposing role to CRISP1 on flagellar waveform. At higher resolution, however, it is clear that CRISP4 plays a defining role in setting amplitude along the entire length of the flagellum, and that both CRISP1 and CRISP4 are required for optimal waveform and power dissipation.

Further, while all three CRISPs play a role in setting sperm POF, CRISP4 has the largest effect and acts upstream, or in a dominant manner, to CRISP1 (Supplementary Figure 5). All three CRISPs also boost power dissipation from the flagellum. The expansion of the CRISP sub-clade from one member in basal vertebrates, to three members in most mammals, and four in numerous rodent species (Abraham and Chandler, 2017; Gaikwad et al., 2020a) thus represents a beautiful example of neofunctionalization wherein proteins with a similar core function (boosting power output) have been tailored to optimize slightly different aspects of sperm tail performance, and enhance reproductive success.

The precise molecular mechanisms by which CRISPs alter axoneme function is unknown. Given that sperm first encounter CRISP1 and CRISP4 during epididymal maturation, a period when sperm are transcriptionally and translationally silent, this is most likely via plasma membrane localized ‘receptors’. This conclusion is supported by the rescue experiments conducted herein wherein the addition of recombinant CRISP1 or CRISP4 to sperm from respective knockout mice resulted in a phenotypic rescue of sperm motility. Similarly, the failure to rescue the phenotype when recombinant CRISP1 was added to sperm from *Crisp4* null mice, and *vice versa*, further indicated that CRISP1 and CRISP4 are functionally distinct and act via different plasma membrane localized receptors. Of interest, while we predict such signaling normally occurs in the epididymis, during epididymal sperm maturation, the data shown here demonstrates that the addition of recombinant CRISPs *in vitro* can compensate, indicating that in this instance the temporal sequence of signaling is not critical. By contrast, based on localization data, CRISP2 within sperm acts in an intra-cellular manner. The identify of these receptors is unknown but can be speculated upon based on previously published data.

For all of the CRISPs we anticipate that at least some of the biological activity is mediated via ion channels based on prior studies demonstrating that ICR domains can regulate ion channel function across a range of species (Gibbs et al., 2006, 2011; Zhou et al., 2008; Wang et al., 2010). Mammalian sperm possess a variety of ion channels, including CATSPER, SLO3, P2X2, TRPM8, and Hv1, all of which are required for optimal sperm motility (Ren et al., 2001; Lishko et al., 2010; Navarro et al., 2011; Chavez et al., 2014). Previous studies have demonstrated that mouse and rat CRISP4 can regulate mouse TRPM8 channels on sperm (Gibbs et al., 2011; Ernesto et al., 2015). TRPM8 channels are localized to the acrosome region and along the length of the sperm tail where they contribute to at least the epididymal maturation of the acrosome (Gibbs et al., 2011; Martínez-López et al., 2011). While not specifically tested here, the highly restrained nature of the flagellar waveform of sperm from *Crisp4* null mice is consistent with the localization of TRPM8. CRISP2 can regulate ryanodine receptors and bind to the CATSPER1 subunit of the CATSPER channel, presumably onto a cytoplasmic surface (Gibbs et al., 2006; Lim et al., 2019). Ryanodine receptors are found in the head-tail coupling region likely in the redundant nuclear envelop, a calcium store in the junction of the sperm head and tail (Harper et al., 2004), and is thus consistent with the stiff mid-piece phenotype observed in sperm from *Crisp2* null mice. CATSPER channels by contrast are localized on the principal piece of the tail and while unlikely to be the mediator of CRISP2 based on the observation that the loss of CRISP2 has the greatest effect on the mid-piece, it may be a “receptor” through which CRISP4 alters axoneme function (Ernesto et al., 2015). No *bona fide* CRISP1 receptors have been identified to date. This, and the mismatch in the CRISP2 localization and null sperm phenotype data, suggests that other as yet unknown CRISP receptors exist on sperm. The identity of these receptors, and the possibility that CAP domain receptors may also exist on sperm, as indicated by the role of non-CRISP CAP proteins on sperm function (Gaikwad et al., 2019), is the subject of ongoing research.

Given the commonality of the known CRISP-regulated channels, and other CRISP-regulated ion channels identified in reptiles (Tadokoro et al., 2020), we predict that CRISPs function to regulate calcium flow from the external environment, in the case of epididymal CRISPs, or intracellular calcium stores in the case of CRISP2. As an extension, we predict that such changes in calcium regulate the POF and power generated by ATP hydrolysis by the inner and outer dynein arms of the axoneme. While we cannot rule out the possibility that CRISPs lead to increased ATP generation and hydrolysis by the inner and outer dynein arms, the data presented here favors a role in coordinating and optimizing microtubule sliding within the axoneme and thus waveform.

Herein we have utilized the planar motion of head-tethered sperm to investigate the role of CRISPs in establishing optimal flagellar beating as it is relevant to the situation in mammals wherein sperm are known to accumulate and swim close to epithelial surfaces and display largely planar motility (Nosrati et al., 2015; Raveshi et al., 2021). Such an analysis would not be appropriate for species where sperm swim freely through aqueous fluids e.g., species where spawning occurs. Moreover, tethering of the sperm head and the relatively narrow depth of the visualization chamber while it may restrain flagella movement in the Z direction does allow for the measurement of biomechanical outputs from the entire sperm tail as well as specific sub-regions. As such, this method may prove of value for the functional assessment of proteins and molecules that are regionally restricted within the tail. For an assessment of free-swimming a three-dimensional analysis is recommended (Daloglu et al., 2018). While beyond the scope of the current system, we are attempting to modify the current low cost imaging system and mathematical analysis pipeline to allow the assessment of free-swimming (head rotating) to allow the reconstruction of flagellar wave in three dimension from 2D dark-field images (Powar et al., 2021).

Similarly, the current method must be used cautiously for the analysis of capacitated sperm or sperm displaying hyperactivated motility. Following a period of maturation in the female reproductive tract mammalian sperm display hyperactivated motility, which is characterized by a high amplitude whip-like waveform wherein the tail frequently crosses itself (Suarez and Ho, 2003). The imaging and mathematical approach outlined here cannot accommodate such crossing events. Previous data has suggested that CRISP1 and 4 play a role in this processes (Nixon et al., 2006; Da Ros et al., 2008; Hu et al., 2018). As such, an imagining system capable of resolving flagellar motion at both high resolution and in three dimensions would be required to address the role of CRISPs, or other molecules, in regulating sperm tail motility.

The mere existence of CRISPs in the male reproductive tract has been a conundrum for many years. They are not required for male fertility, yet they are incorporated into sperm, and bathe, sperm at high concentrations across mammalian species (Gaikwad et al., 2020a). As indicated here, and as posited previously (Hu et al., 2018; Vicens and Treviño, 2018), we hypothesize this is due to the competitive advantage CRISPs confer upon sperm in situations of competition. While not normally the case in research facilities, mice are polyandrous in the wild. Females mate with multiple males in quick succession and sperm compete for oocyte fertilization (Rolland et al., 2003; Firman and Simmons, 2008). Many genes/proteins involved in conferring reproductive benefit are known to undergo adaptive evolution (positive Darwinian evolution). This is driven by gene duplication and higher rates of genetic change, leading to amino acid substitutions, more often than in most other genes (Swanson and Vacquier, 2002). Such rapidly evolving genes are thought to play key roles in optimizing sperm motility, in conspecific compatibility in key fertilization proteins and ultimately speciation (Swanson et al., 2001; Vicens et al., 2014a). Studies have shown that mammalian CRISP genes have experienced positive evolution in both the CAP and CRISP domains (Vicens and Treviño, 2018; Arévalo et al., 2020). Cross-species sequence comparison indicate that *Crisp2* is likely the ancestral gene from which *Crisp1* was derived (Arévalo et al., 2020; Gaikwad et al., 2020a). *Crisp1* has then undergone rapid sequence divergence within rodents, and a further duplication to produce *Crisp4*. Of particular interest, evolutionary analysis, reveal that higher rates of sperm energy consumption, a surrogate of power output, is associated with the occurrence of sperm competition within a species, and higher and more efficient sperm velocity (Tourmente et al., 2019). Data presented here reveal CRISPs confer such an effect.

As outlined above, the ICR domain is known to regulate a range of key ion channels required for sperm function. Several of these ion channels, including *CatSper1* (Vicens et al., 2014b) and TRPM8 (Majhi et al., 2015), have also experienced positive selection. In addition, the CAP domains, including those in CRISPs, are proposed to have roles in sperm-oocyte fusion (Yamaguchi et al., 2011; Ernesto et al., 2015; Gaikwad et al., 2019). These data suggest that the recruitment of an ICR domain onto an ancestral CAP domain, and hinge region, during evolution to form the founding CRISP gene in vertebrates, then their subsequent expansion and over expression within the mammalian male reproductive tract (Abraham and Chandler, 2017), represents an example of positive Darwinian evolution driven by the combined ability of CRISPs to boost sperm motility and assist in oocyte binding. Collectively, these data support the hypothesis, that CRISPs have been acquired and expanded during the processes of evolution as a consequence of their beneficial role on sperm function and male reproductive success in situations of inter-male competition.

## Data Availability Statement

The datasets presented in this study can be found in online repositories. The names of the repository/repositories and accession number(s) can be found in the article/ Supplementary Material.

## Ethics Statement

The animal study was reviewed and approved by Monash University Biological Sciences Animal Experimentation Ethics Committee. Written informed consent was obtained from the owners for the participation of their animals in this study.

## Author Contributions

MO’B and AG designed the research and drafted the manuscript. DP, AN, AG, and RP were involved in the development of essential analysis tools. All authors were involved in data analysis and editing of the manuscript.

## Conflict of Interest

The authors declare that the research was conducted in the absence of any commercial or financial relationships that could be construed as a potential conflict of interest.

## Publisher’s Note

All claims expressed in this article are solely those of the authors and do not necessarily represent those of their affiliated organizations, or those of the publisher, the editors and the reviewers. Any product that may be evaluated in this article, or claim that may be made by its manufacturer, is not guaranteed or endorsed by the publisher.

## Footnotes

1
https://fiji.sc

2
https://micro-manager.org

3
https://doi.org/10.26180/5ebb4bf41c4eb

4
https://doi.org/10.26180/5dbf4bff1acce

## Supplementary Material

Supplementary Figure 1 Assessment of recombinant mouse CRISP1 protein by **(A)** SDS-PAGE gel (12%) stained with Coomassie blue and **(B)** western blot analysis using a CRISP1 antibody. Molecular weight (kDa) is indicated on the left-hand side. F1 and F2 correspond to pooled and concentrated sample loaded in duplicate. Red arrows indicate recombinant mouse CRISP1. The two bands correspond to differentially glycosylated forms as characterized previously (Volpert et al., 2014).

Supplementary Figure 2 Effect of time on sperm motility parameter. The (POF) of wild type sperm as a function of time post back-flushing. A minimum of 5 sperm from 3 adult wild type mice (C57BL/6JxC57BL/6N) were analyzed, *N* = 15 sperm in total. *ns* indicates no significance and ^∗^ indicates *P* < 0.01 as analyzed by one-way ANOVA.

Supplementary Figure 3 Proper orthogonal decomposition (POD) analysis of sperm flagellar beating. **(A)** The percentage contribution of individual shape mode vectors ψ_1_, ψ_2_, ψ_3_, and ψ_4_ across sperm from wild type and *Crisp* knock out mice. **(B)** The four averaged shape modes across sperm from wild type and *Crisp* knock out mice. The gray lines indicate the modes of individual sperm and the bold line indicates averaged shape mode across the sperm population.

Supplementary Figure 4 LIVE/DEAD staining analysis of sperm from *Crisp1/4* mice colonies. **(A)** Percentage of motile sperm, which were observed to be in motion, from *Crisp1/4* wild type (WT; orange) and knock out (KO; light orange) mice measured at 0 to 60 min post-retrieval from the cauda epididymis and staining with LIVE/DEAD^TM^ staining kit. **(B)** Percentage of live-stained sperm, which could be motile or immotile but viable, from *Crisp1/4* wild type (orange) and knock out (light orange) mice measured at intervals from 0 to 60 min. A minimum of 200 sperm were measured from each mouse (*N* = 3 mice/genotype). Different super-script letter indicates statistically significant differences between groups. *P* < 0.0001 as analyzed by two-way ANOVA.

Supplementary Figure 5 Statistical analysis of investigated sperm motility parameters. Sperm from *Crisp* wild type (dotted blue line) and knock out (bold red line) mice strains were evaluated for **(A)** flagellar amplitude along different regions of the flagella, **(B)** power dissipation along different regions of the flagella, **(C)** POF, **(D)** net power dissipated (10^–12^ J/s), and **(E)** projected net progressive velocity. Statistical analysis was performed using ANOVA followed by *post-hoc t*-tests to evaluate the combinations which drove the interactions and main effects. Data presented is from a total of 25–30 sperm/genotype. *N* = 5–6 mice/genotype. Data presented as mean ± SD.

Supplementary Figure 6 Effects of 1 μM recombinant CRISP proteins on the calculated primary oscillating frequency (POF) for sperm from wild type and *Crisp* knock out mice. **(A)** The data presented is 10–15 sperm/genotype. Different letters indicate significant differences between groups, where significance is measured relative to the wild type (black bar). *P* < 0.01 for all marked “b”; and *P* < 0.001 for all marked “c” as determined using a two-way ANOVA. The light red color bars represent *Crisp1* knock out mice data and light red color with dotted bar represent *Crisp1* knock out mice after exposure to recombinant CRISP1 protein. The light yellow color bars represent *Crisp4* knock out mice data and light yellow color with dotted bar represent *Crisp4* knock out mice after exposure to recombinant CRISP4 protein. **(B)** The data presented is 10–15 sperm/genotype. Different letters indicate significant differences between groups, where significance is measured relative to the wild type (black bar). *P* < 0.01 for all marked “b”; and *P* < 0.001 for all marked “c” as determined using a two-way ANOVA. The light red color bars represent *Crisp1* knock out mice data and light red color with yellow dotted bar represent *Crisp1* knock out mice after exposure to recombinant CRISP4 protein. The light yellow color bars represent *Crisp4* knock out mice data and light yellow color with red dotted bar represent *Crisp4* knock out mice after exposure to recombinant CRISP1 protein. **(C)** The data presented is 10–15 sperm/genotype. Different letters indicate significant differences between groups, where significance is measured relative to the *Crisp1/4* wild type (intense orange bar). *P* < 0.01 for all marked “b” as determined using a two-way ANOVA. The light orange color bar represent *Crisp1/4* knock out mice data and light orange color with red dotted bar represent *Crisp1/4* knock out mice after exposure to recombinant CRISP1 protein. The light orange color with yellow dotted bar represent *Crisp1/4* knock out mice after exposure to recombinant CRISP4 protein. The gray color bar represent *Crisp1/4* knock out mice after combined exposure to recombinant CRISP1 and CRISP4 protein.

Supplementary Figure 7 Effects of 1 μM recombinant CRISP4 protein on sperm from *Crisp1* knock out mice and *vice versa*. **(A)** The representative shape cycles of the flagellar beating, reconstruction of mean beat cycle and analysis of power dissipation (10^–8^ W/m) along the flagella of the sperm from *Crisp1* (red) and *Crisp4* (black) knock out mice before (solid bar) and after (shaded bar) exposure to recombinant CRISP4 and CRISP1 protein, respectively. *N* = 10–15 sperm/genotype were analyzed, and representative mean beat cycle and shape cycles of the flagellar beating is shown. For power dissipation, the black bold line indicates averaged data across the analyzed sperm population ± SEM. **(B)** Analysis of flagellar amplitude along the different regions of flagella (mid-piece, principal piece, and end piece) in sperm from *Crisp1* and *Crisp4* knock out mice before and after exposure to recombinant CRISP4 and CRISP1 protein, respectively. **(C)** Analysis of power dissipation (10^–12^ J/s) along the different regions of flagella (mid-piece, principal piece, and end piece) in sperm from *Crisp1* and *Crisp4* knock out mice before and after exposure to recombinant CRISP4 and CRISP1 protein, respectively. The data presented is from a total number of 10–15 sperm/genotype. ^****^ indicates *P* < 0.0001 as analyzed by two-way ANOVA ± SEM. See Supplementary Tables 9.1, 9.2 for all the possible interactions between exposed and non-exposed genotypes.

Supplementary Figure 8 Effects of recombinant epididymal CRISPs on sperm from wild type and *Crisp1*/*4* double knock out mice. **(A)** The representative shape cycles of the flagellar beating and reconstruction of mean beat cycle of the sperm from *Crisp1/4* wild type (black) and knock out (orange) mice before and after exposure to individual recombinant CRISP1 (red), CRISP4 (yellow) and combined CRISP1 and CRISP4 (light orange) proteins. *N* = 10–15 sperm/genotype were analyzed, and representative mean beat cycle and shape cycles of the flagellar beating is shown. **(B)** Analysis of flagellar amplitude along the different regions of flagella (mid-piece, principal piece, and end piece) in sperm from *Crisp1/4* knock out mice before and after exposure to recombinant epididymal CRISPs, respectively. **(C)** Analysis of power dissipation (10^–12^ J/s) along the different regions of flagella (mid-piece, principal piece and end piece) in sperm from *Crisp1/4* knock out mice before and after exposure to recombinant epididymal CRISPs. The data presented is from a total number of 10–15 sperm/genotype. ^****^ indicates *P* < 0.0001 as analyzed by two-way ANOVA ± SEM.

Supplementary Video 1 Representative video of head tethered sperm from *Crisp1* wild type mice. Sperm were tethered onto a glass slides (as described in section “Materials and Methods”) and recorded at 400 fps using an Olympus AX-70 upright microscope equipped with ORCA-Flash4.0 v2 sCMOS camera. The primary oscillating frequency (POF) for presented sperm is 7.3 Hz.

Supplementary Video 2 Representative video of head tethered sperm from *Crisp2* wild type mice. The imaging was performed as described for Supplementary Video 1 and in section “Materials and Methods.” The POF for presented sperm is 7.7 Hz.

Supplementary Video 3 Representative video of head tethered sperm from *Crisp4* wild type mice. The imaging was performed as described for Supplementary Video 1 and in section “Materials and Methods.” The POF for presented sperm is 7.1 Hz.

Supplementary Video 4 Representative video of head tethered sperm from *Crisp1/4* wild type mice. The imaging was performed as described for Supplementary Video 1 and in section “Materials and Methods.” The POF for presented sperm is 7.3 Hz.

Supplementary Video 5 Representative video of head tethered sperm from *Crisp1* knock out mice. The imaging was performed as described for Supplementary Video 1 and in section “Materials and Methods.” The POF for presented sperm is 5.9 Hz.

Supplementary Video 6 Representative video of head tethered sperm from *Crisp2* knock out mice. The imaging was performed as described for Supplementary Video 1 and in section “Materials and Methods”. The POF for presented sperm is 5.6 Hz.

Supplementary Video 7 Representative video of head tethered sperm from *Crisp4* knock out mice. The imaging was performed as described for Supplementary Video 1 and in section “Materials and Methods.” The POF for presented sperm is 4.7 Hz.

Supplementary Video 8 Representative video of head tethered sperm from *Crisp1/4* knock out mice. The imaging was performed as described for Supplementary Video 1 and in section “Materials and Methods.” The POF for presented sperm is 4.8 Hz.

Supplementary Video 9 Representative video of head tethered sperm from *Crisp1* wild type mice after exposure to 1 μM recombinant CRISP1 protein. The imaging was performed as described for Supplementary Video 1 and in section “Materials and Methods.” The POF for presented sperm is 7.2 Hz.

Supplementary Video 10 Representative video of head tethered sperm from *Crisp4* wild type mice after exposure to 1 μM recombinant CRISP4 protein. The imaging was performed as described for Supplementary Video 1 and in section “Materials and Methods.” The POF for presented sperm is 7.3 Hz.

Supplementary Video 11 Representative video of head tethered sperm from *Crisp1* knock out mice after exposure to 1 μM recombinant CRISP1 protein. The imaging was performed as described for Supplementary Video 1 and in section “Materials and Methods.” The POF for presented sperm is 7.6 Hz.

Supplementary Video 12 Representative video of head tethered sperm from *Crisp4* knock out mice after exposure to 1 μM recombinant CRISP4 protein. The imaging was performed as described for Supplementary Video 1 and in section “Materials and Methods.” The POF for presented sperm is 7.5 Hz.
