# Supplementary material for: CRISPs Function to Boost Sperm Power Output and Motility
Source: Front Cell Dev Biol. 2021 Aug 5;9:693258. doi: 10.3389/fcell.2021.693258 (PMC8374954; doi:10.3389/fcell.2021.693258)
Supplement: Supplementary Figure 1 — Assessment of recombinant mouse CRISP1 protein by (A) SDS-PAGE gel (12%) stained with Coomassie blue and (B) western blot analysis using a CRISP1 antibody. Molecular weight (kDa) is indicated on the left-hand side. F1 and F2 correspond to pooled and concentrated sample loaded in duplicate. Red arrows indicate recombinant mouse CRISP1. The two bands correspond to differentially glycosylated forms as characterized previously (Volpert et al., 2014). [file Data_Sheet_1.pdf]

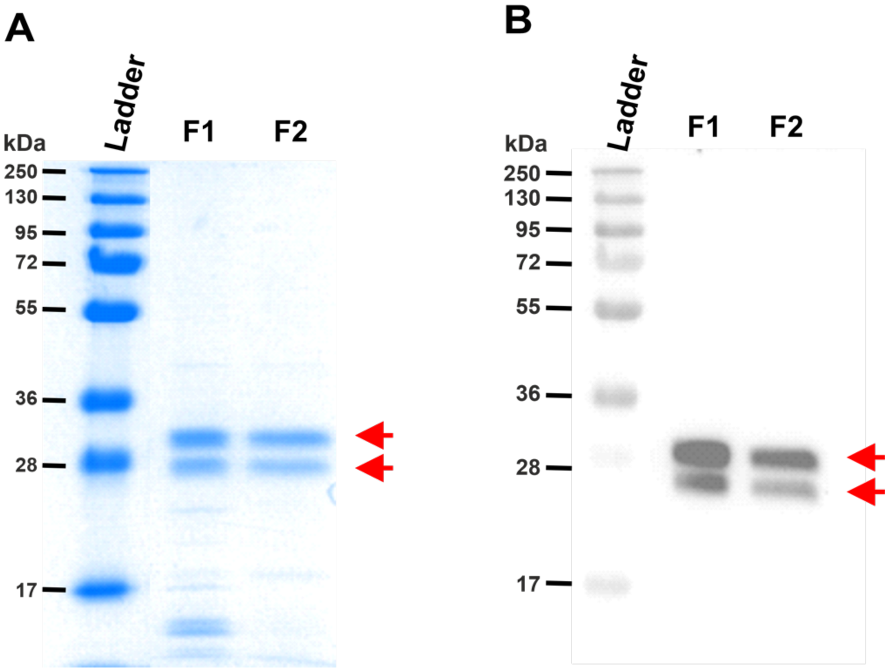

Supplementary Figure 1. Assessment of recombinant mouse CRISP1 protein by (A) SDS-PAGE gel (12%) stained with Coomassie blue and (B) western blot analysis using a CRISP1 antibody. Molecular weight (kDa) is indicated on the left-hand side. F1 and F2 correspond to pooled and concentrated sample loaded in duplicate. Red arrows indicate recombinant mouse CRISP1 protein. The two bands correspond to differentially glycosylated forms as characterized previously (Volpert et al., 2014).

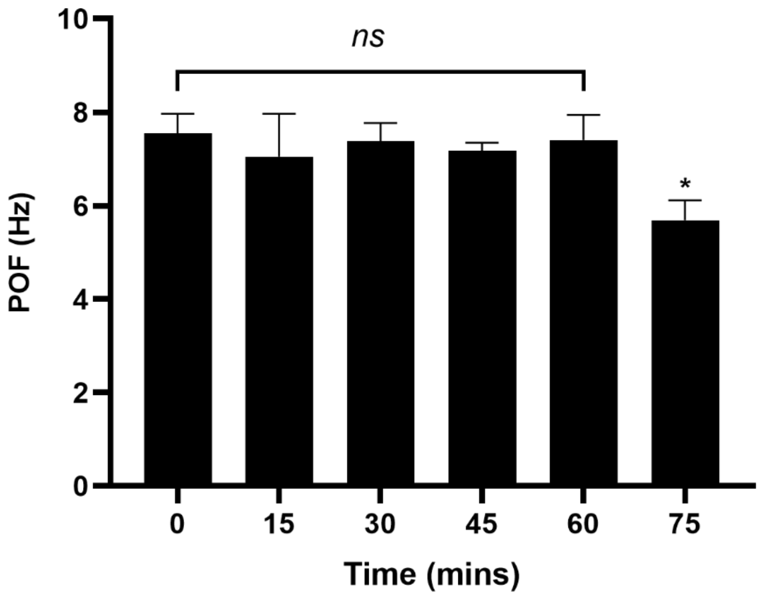

Supplementary Figure 2. Effect of time on sperm motility parameter. The (POF) of wild type sperm as a function of time post back-flushing. A minimum of 5 sperm from 3 adult wild type mice (C57BL/6JxC57BL/6N) were analysed, N = 15 sperm in total. *ns* indicates no significance and * indicates $P < 0.01$ as analyzed by one-way ANOVA.

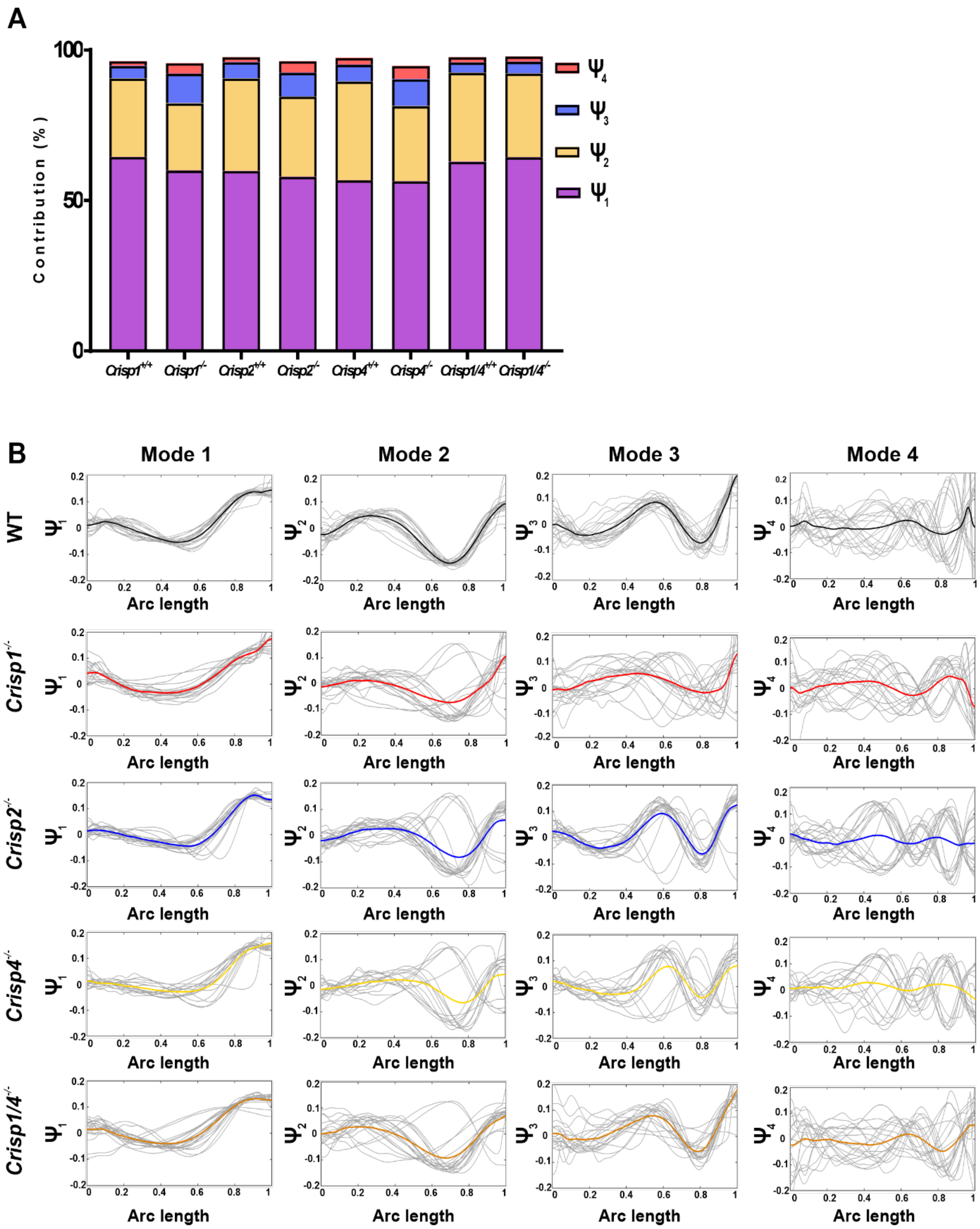

Supplementary Figure 3. Proper orthogonal decomposition (POD) analysis of sperm flagellar beating. A) The percentage contribution of individual shape mode vectors ψ_1, ψ_2, ψ_3 and ψ_4 across sperm from wild type and *Crisp* knock out mice. **B)** The four averaged shape modes across sperm from wild type and *Crisp* knock out mice. The gray lines indicate

the modes of individual sperm and the bold line indicates averaged shape mode across the sperm population.

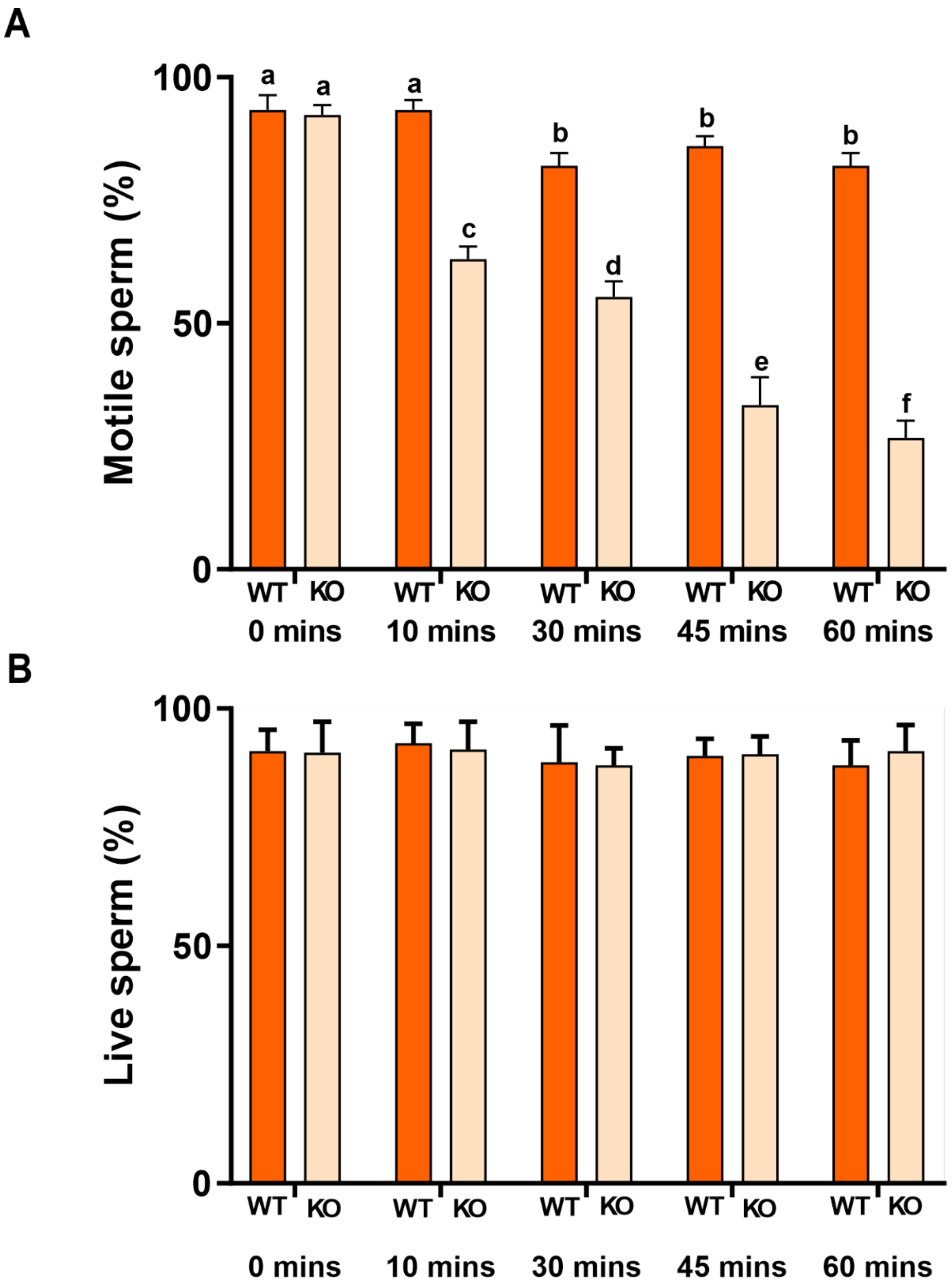

Supplementary Figure 4: LIVE/DEAD staining analysis of sperm from *Crisp1/4* mice colonies. A) Percentage of motile sperm, which were observed to be in motion, from *Crisp1/4* wild type (WT; orange) and knock out (KO; light orange) mice measured at 0 to 60 mins post-retrieval from the cauda epididymis and staining with LIVE/DEAD™ staining kit. **B)** Percentage of live-stained sperm, which could be motile or immotile but viable, from *Crisp1/4* wild type (orange) and knock out (light orange) mice measured at intervals from 0 to 60 mins. A minimum of 200 sperm were measured from each mouse (N = 3 mice/genotype). Different super-script letter indicates statistically significant differences between groups. $P < 0.0001$ as analyzed by two-way ANOVA.

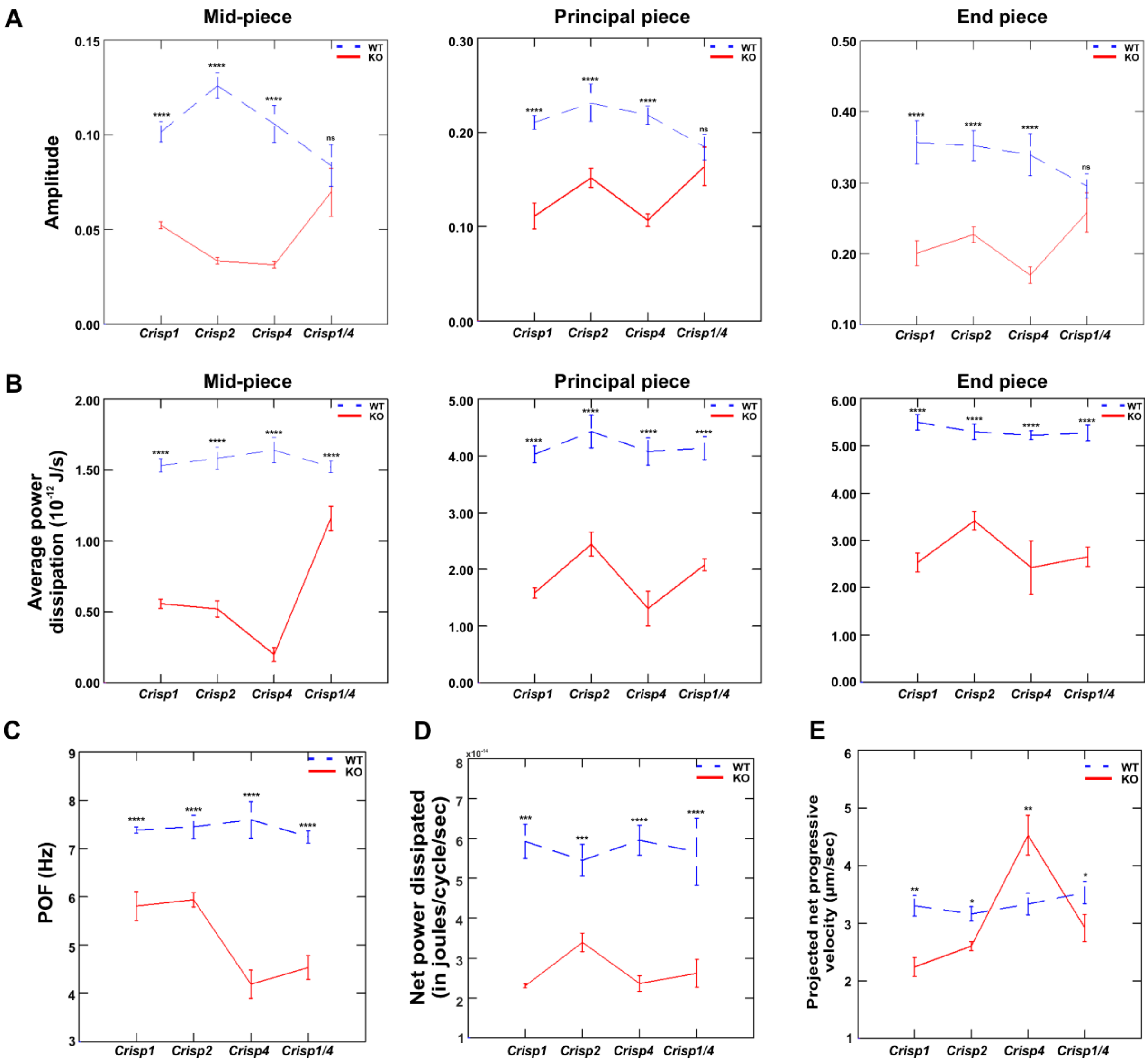

Supplementary Figure 5: Statistical analysis of investigated sperm motility parameters. Sperm from *Crisp* wild type (dotted blue line) and knock out (bold red line) mice strains were evaluated for **(A)** flagellar amplitude along different regions of the flagella, **(B)** power dissipation along different regions of the flagella, **(C)** POF, **(D)** net power dissipated (10^{-12} J/s) and **(E)** projected net progressive velocity. Statistical analysis was performed using ANOVA followed by *post-hoc* t-tests to evaluate the combinations which drove the interactions and main effects. Data presented is from a total of 25-30 sperm/genotype. *N* = 5-6 mice/genotype. Data presented as mean ± S.D.

A

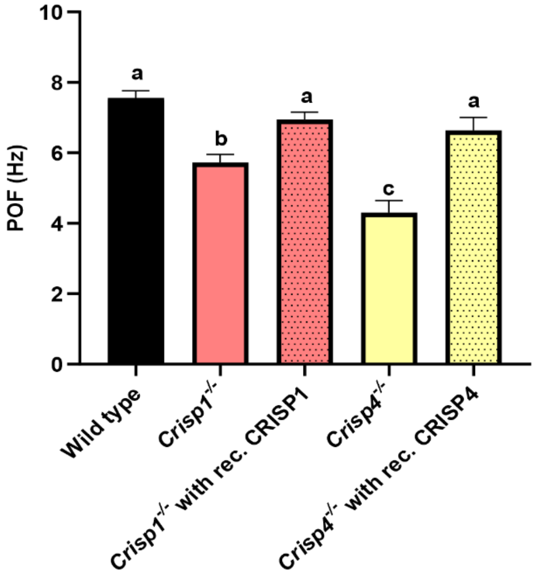

POF (Hz)
10
8
6
4
2
0
a
b
a
c
a
Wild type
Crisp1-/-
Crisp1-/- with rec. CRISP1
Crisp4-/-
Crisp4-/- with rec. CRISP4

B

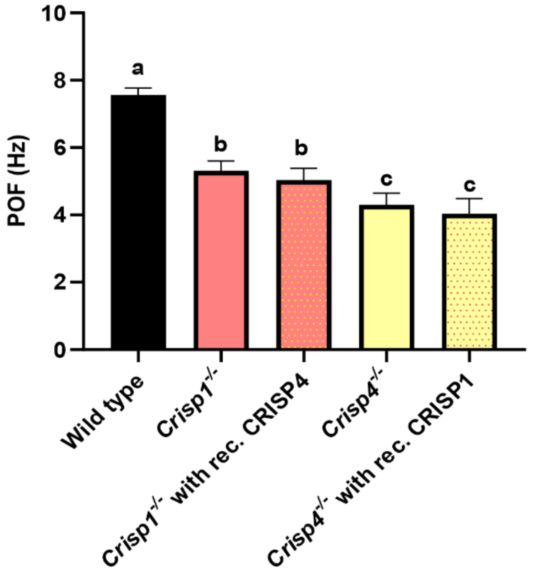

POF (Hz)
10
8
6
4
2
0
a
b
b
c
c
Wild type
Crisp1-/-
Crisp1-/- with rec. CRISP4
Crisp4-/-
Crisp4-/- with rec. CRISP1

C

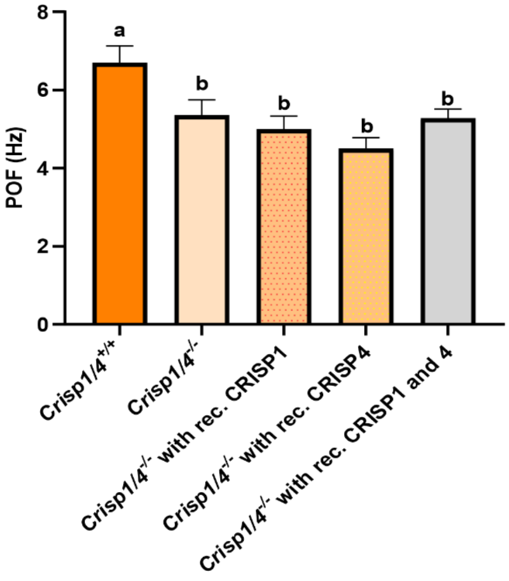

POF (Hz)
8
6
4
2
0
a
b
b
b
b
Crisp1/4+/+
Crisp1/4-/-
Crisp1/4-/- with rec. CRISP1
Crisp1/4-/- with rec. CRISP4
Crisp1/4-/- with rec. CRISP1 and 4

Supplementary Figure 6. Effects of 1 µM recombinant CRISP proteins on the calculated primary oscillating frequency (POF) for sperm from wild type and *Crisp* knock out mice. **A)** The data presented is 10-15 sperm/genotype. Different letters indicate significant differences between groups, where significance is measured relative to the wild type (black bar). $P < 0.01$ for all marked 'b'; and $P < 0.001$ for all marked 'c' as determined using a two-way ANOVA. The light red colour bars represent *Crisp1* knock out mice data and light red colour with dotted bar represent *Crisp1* knock out mice after exposure to recombinant CRISP1 protein. The light yellow colour bars represent *Crisp4* knock out mice data and light yellow colour with dotted bar represent *Crisp4* knock out mice after exposure to recombinant CRISP4 protein. **B)** The data presented is 10-15 sperm/genotype. Different letters indicate significant differences between groups, where significance is measured relative to the wild type (black bar). $P < 0.01$ for all marked 'b'; and $P < 0.001$ for all marked 'c' as determined using a two-way ANOVA. The light red colour bars represent *Crisp1* knock out mice data and light red colour with yellow dotted bar represent *Crisp1* knock out mice after exposure to recombinant CRISP4 protein. The light yellow colour bars represent *Crisp4* knock out mice data and light yellow colour with red dotted bar represent *Crisp4* knock out mice after exposure to recombinant CRISP1 protein. **C)** The data presented is 10-15 sperm/genotype. Different letters indicate significant differences between groups, where significance is measured relative to the *Crisp1/4* wild type (intense orange bar). $P < 0.01$ for all marked 'b' as determined using a two-way ANOVA. The light orange colour bar represent *Crisp1/4* knock out mice data and light orange colour with red dotted bar represent *Crisp1/4* knock out mice after exposure to recombinant CRISP1 protein. The light orange colour with yellow dotted bar represent *Crisp1/4* knock out mice after exposure to recombinant CRISP4 protein. The gray colour bar represent *Crisp1/4* knock out mice after combined exposure to recombinant CRISP1 and CRISP4 protein.

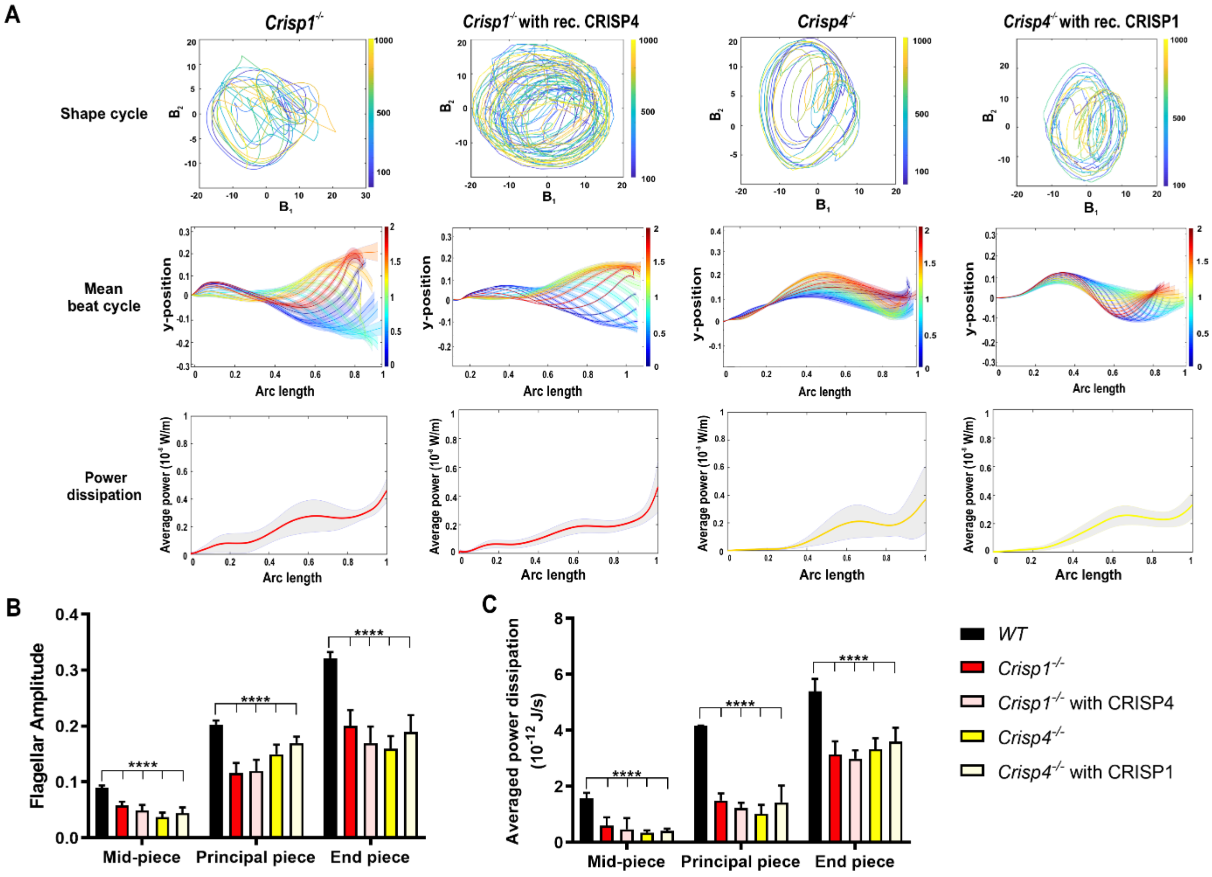

Supplementary Figure 7. Effects of 1 µM recombinant CRISP4 protein on sperm from *Crisp1* knock out mice and *vice versa*. A) The representative shape cycles of the flagellar beating, reconstruction of mean beat cycle and analysis of power dissipation (10^{-8} W/m) along the flagella of the sperm from *Crisp1* (red) and *Crisp4* (black) knock out mice before (solid bar) and after (shaded bar) exposure to recombinant CRISP4 and CRISP1 protein, respectively. *N* = 10-15 sperm/genotype were analyzed, and representative mean beat cycle and shape cycles of the flagellar beating is shown. For power dissipation, the black bold line indicates averaged data across the analyzed sperm population ± SEM. **B)** Analysis of flagellar amplitude along the different regions of flagella (mid-piece, principal piece and end piece) in sperm from *Crisp1* and *Crisp4* knock out mice before and after exposure to recombinant CRISP4 and CRISP1 protein, respectively. **C)** Analysis of power dissipation (10^{-12} J/s) along the different regions of

flagella (mid-piece, principal piece and end piece) in sperm from *Crisp1* and *Crisp4* knock out mice before and after exposure to recombinant CRISP4 and CRISP1 protein, respectively. The data presented is from a total number of 10-15 sperm/genotype. **** indicates $P < 0.0001$ as analyzed by two-way ANOVA ± S.E.M. See Supplementary Table 9.1 and 9.2 for all the possible interactions between exposed and non-exposed genotypes.

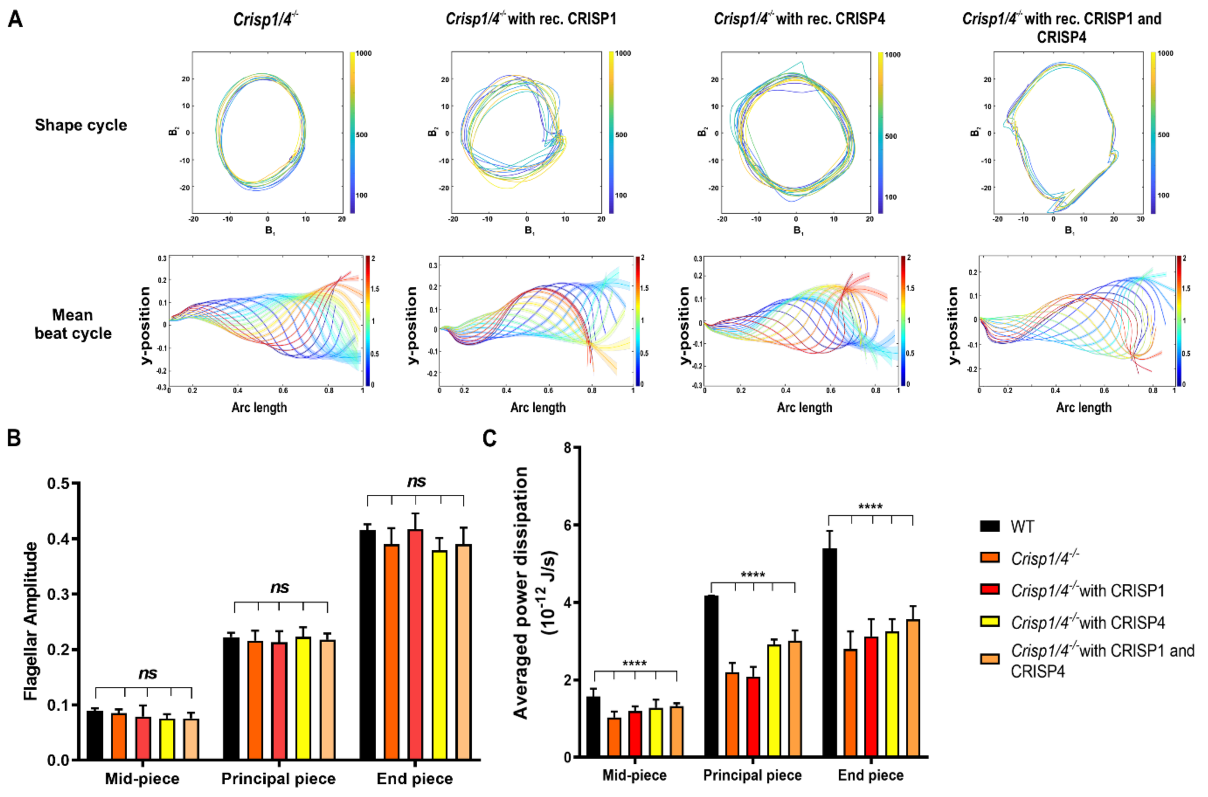

Supplementary Figure 8: Effects of recombinant epididymal CRISPs on sperm from wild type and *Crisp1/4* double knock out mice. A) The representative shape cycles of the flagellar beating and reconstruction of mean beat cycle of the sperm from *Crisp1/4* wild type (black) and knock out (orange) mice before and after exposure to individual recombinant CRISP1 (red), CRISP4 (yellow) and combined CRISP1 and CRISP4 (light orange) proteins. $N = 10\text{-}15$ sperm/genotype were analyzed, and representative mean beat cycle and shape cycles of the flagellar beating is shown. B) Analysis of flagellar amplitude along the different regions of flagella (mid-piece, principal piece and end piece) in sperm from *Crisp1/4* knock out mice

before and after exposure to recombinant epididymal CRISPs, respectively. **C)** Analysis of power dissipation (10^{-12} J/s) along the different regions of flagella (mid-piece, principal piece and end piece) in sperm from *Crisp1/4* knock out mice before and after exposure to recombinant epididymal CRISPs. The data presented is from a total number of 10-15 sperm/genotype. **** indicates $P < 0.0001$ as analyzed by two-way ANOVA ± S.E.M.
