# Supplementary material for: CRISPs Function to Boost Sperm Power Output and Motility
Source: Front Cell Dev Biol. 2021 Aug 5;9:693258. doi: 10.3389/fcell.2021.693258 (PMC8374954; doi:10.3389/fcell.2021.693258)
Supplement: Supplementary file 16 [file Data_Sheet_2.docx]

**Supplementary Table 1.** The calculated Procrustes measure of flagellar beat across wild type and respective knock out mice lines.

| **Wild type combinations** | **Procrustes measure ± SD** | ***P* value** |
| --- | --- | --- |
| *Crisp1^+/+^ vs Crisp2^+/+^* | 3.0 ± 1.75 | ns |
| *Crisp1^+/+^* vs *Crisp4^+/+^* | 3.5 ± 1.9 | ns |
| *Crisp1^+/+^* vs *Crisp1/4^+/+^* | 3.1 ± 1.7 | ns |
| *Crisp2^+/+^* vs *Crisp4^+/+^* | 3.1 ± 1.7 | ns |
| *Crisp2^+/+^* vs *Crisp1/4^+/+^* | 3.7 ± 1.9 | ns |
| *Crisp4^+/+^* vs *Crisp1/4^+/+^* | 3.99 ± 1.90 | ns |

**Supplementary Table 2.** The calculated primary oscillating frequency (in Hz) across sperm from wild type and *Crisp* knock out mice.

| **Mouse strain** | **Wild type ± SD** | **Knock out ± SD** | **P value** |
| --- | --- | --- | --- |
| CRISP1 | 7.3 ± 0.13 | 5.8 ± 0.65 | < 0.05 |
| CRISP2 | 7.4 ± 0.53 | 5.9 ± 0.32 | < 0.05 |
| CRISP4 | 7.5 ± 0.83 | 4.1 ± 0.64 | <0.0001 |
| CRISP1/4 | 7.2 ± 0.28 | 4.8 ± 0.34 | <0.0001 |

**Supplementary Table 3.** Analysis of flagellar amplitude at different regions of flagella in the presence or absence of *Crisp* genes.

| **Region on the flagella** | ***Crisp1*** | | **P Value** | ***Crisp2*** | | **P Value** | ***Crisp4*** | | **P Value** | ***Crisp1/4*** | | **P Value** |
| --- | --- | --- | --- | --- | --- | --- | --- | --- | --- | --- | --- | --- |
|  | **WT** | **KO** |  | **WT** | **KO** |  | **WT** | **KO** |  | **WT** | **KO** |  |
| Mid-piece | 0.10 ± 0.24 | 0.05 ± 0.009 | 0.0222 | 0.12 ± 0.03 | 0.03 ± 0.01 | <0.0001 | 0.10 ± 0.03 | 0.03 ± 0.08 | <0.0001 | 0.11 ± 0.03 | 0.12 ± 0.04 | 0.9982 |
| Principal piece | 0.21 ± 0.03 | 0.11 ± 0.05 | <0.0001 | 0.22 ± 0.05 | 0.15 ± 0.04 | <0.0001 | 0.21 ± 0.04 | 0.10 ± 0.03 | <0.0001 | 0.23 ± 0.04 | 0.24 ± 0.06 | 0.9999 |
| End piece | 0.35 ± 0.08 | 0.196 ± 0.06 | <0.0001 | 0.34 ± 0.07 | 0.22 ± 0.08 | <0.0001 | 0.33 ± 0.08 | 0.17 ± 0.07 | <0.0001 | 0.35 ± 0.07 | 0.35 ± 0.09 | 0.9999 |
| *Please refer to Supplementary Table 3.1 for all the possible interactions between wild type vs. knock out genotype* | | | | | | | | | | | | |

**Supplementary Table 3.1.** Analysis of flagellar amplitude along the different regions of flagella across *Crisp* wild type and knock out mice combinations.

|  | ***Crisp1*-WT** | **C*risp1*-KO** | ***Crisp2*-WT** | ***Crisp2*-KO** | ***Crisp4*-WT** | ***Crisp4*-KO** | ***Crisp1/4*-WT** | ***Crisp1/4*-KO** | ***Crisp1*-WT** | **C*risp1*-KO** | ***Crisp2*-WT** | ***Crisp2*-KO** | ***Crisp4*-WT** | ***Crisp4*-KO** | ***Crisp1/4*-WT** | ***Crisp1/4*-KO** | ***Crisp1*-WT** | **C*risp1*-KO** | ***Crisp2*-WT** | ***Crisp2*-KO** | ***Crisp4*-WT** | ***Crisp4*-KO** | ***Crisp1/4*-WT** | ***Crisp1/4*-KO** |
| --- | --- | --- | --- | --- | --- | --- | --- | --- | --- | --- | --- | --- | --- | --- | --- | --- | --- | --- | --- | --- | --- | --- | --- | --- |
| ***Crisp1*-WT** |  | 0.022 | 0.7295 | 0.0001 | >0.9999 | <0.0001 | 0.9366 | 0.6 |  | <0.0001 | 0.9208 | 0.0021 | 0.9995 | <0.0001 | 0.7063 | 0.4073 |  | <0.0001 | 0.9998 | <0.0001 | 0.9397 | <0.0001 | >0.9999 | >0.9999 |
| ***Crisp1*-KO** | 0.022 |  | <0.0001 | 0.9123 | 0.0081 | 0.856 | 0.0002 | <0.0001 | <0.0001 |  | <0.0001 | 0.13 | <0.0001 | >0.9999 | <0.0001 | <0.0001 | <0.0001 |  | <0.0001 | 0.7739 | <0.0001 | 0.3601 | <0.0001 | <0.0001 |
| ***Crisp2*-WT** | 0.7295 | <0.0001 |  | <0.0001 | 0.8828 | <0.0001 | 0.9998 | >0.9999 | 0.9208 | <0.0001 |  | <0.0001 | 0.9967 | <0.0001 | 0.9999 | 0.9894 | 0.9998 | <0.0001 |  | <0.0001 | 0.9966 | <0.0001 | >0.9999 | >0.9999 |
| ***Crisp2*-KO** | 0.0001 | 0.9123 | <0.0001 |  | <0.0001 | >0.9999 | <0.0001 | <0.0001 | 0.0021 | 0.13 | <0.0001 |  | 0.0002 | 0.0555 | <0.0001 | <0.0001 | <0.0001 | 0.7739 | <0.0001 |  | <0.0001 | 0.0045 | <0.0001 | <0.0001 |
| ***Crisp4*-WT** | >0.9999 | 0.0081 | 0.8828 | <0.0001 |  | <0.0001 | 0.9871 | 0.7888 | 0.9995 | <0.0001 | 0.9967 | 0.0002 |  | <0.0001 | 0.9474 | 0.76 | 0.9397 | <0.0001 | 0.9966 | <0.0001 |  | <0.0001 | 0.9531 | 0.9722 |
| ***Crisp4*-KO** | <0.0001 | 0.856 | <0.0001 | >0.9999 | <0.0001 |  | <0.0001 | <0.0001 | <0.0001 | >0.9999 | <0.0001 | 0.0555 | <0.0001 |  | <0.0001 | <0.0001 | <0.0001 | 0.3601 | <0.0001 | 0.0045 | <0.0001 |  | <0.0001 | <0.0001 |
| ***Crisp1/4*-WT** | 0.9366 | 0.0002 | 0.9998 | <0.0001 | 0.9871 | <0.0001 |  | 0.9982 | 0.7063 | <0.0001 | 0.9999 | <0.0001 | 0.9474 | <0.0001 |  | 0.9999 | >0.9999 | <0.0001 | >0.9999 | <0.0001 | 0.9531 | <0.0001 |  | 0.9999 |
| ***Crisp1/4*-KO** | 0.6 | <0.0001 | >0.9999 | <0.0001 | 0.7888 | <0.0001 | 0.9982 |  | 0.4073 | <0.0001 | 0.9894 | <0.0001 | 0.76 | <0.0001 | 0.9999 |  | >0.9999 | <0.0001 | >0.9999 | <0.0001 | 0.9722 | <0.0001 | 0.9999 |  |
|  | **Mid-piece** | | | | | | | | **Principal piece** | | | | | | | | **End piece** | | | | | | | |

**the shaded cells indicate statistically non-significant interactions* *between wild type combinations*.

**Supplementary Table 4.** Analysis of power dissipation per beat cycle along the different regions of flagella across *Crisp* wild type and knock out mice combinations.

|  | ***Crisp1*-WT** | **C*risp1*-KO** | ***Crisp2*-WT** | ***Crisp2*-KO** | ***Crisp4*-WT** | ***Crisp4*-KO** | ***Crisp1/4*-WT** | ***Crisp1/4*-KO** | ***Crisp1*-WT** | **C*risp1*-KO** | ***Crisp2*-WT** | ***Crisp2*-KO** | ***Crisp4*-WT** | ***Crisp4*-KO** | ***Crisp1/4*-WT** | ***Crisp1/4*-KO** | ***Crisp1*-WT** | **C*risp1*-KO** | ***Crisp2*-WT** | ***Crisp2*-KO** | ***Crisp4*-WT** | ***Crisp4*-KO** | ***Crisp1/4*-WT** | ***Crisp1/4*-KO** |
| --- | --- | --- | --- | --- | --- | --- | --- | --- | --- | --- | --- | --- | --- | --- | --- | --- | --- | --- | --- | --- | --- | --- | --- | --- |
| ***Crisp1*-WT** |  | <0.0001 | >0.9999 | <0.0001 | 0.999 | <0.0001 | >0.9999 | 0.3411 |  | <0.0001 | 0.3866 | <0.0001 | >0.9999 | <0.0001 | 0.999 | <0.0001 |  | <0.0001 | 0.8819 | <0.0001 | 0.8056 | <0.0001 | 0.9258 | <0.0001 |
| ***Crisp1*-KO** | <0.0001 |  | <0.0001 | >0.9999 | <0.0001 | >0.9999 | <0.0001 | 0.0467 | <0.0001 |  | <0.0001 | <0.0001 | <0.0001 | <0.0001 | <0.0001 | 0.3263 | <0.0001 |  | <0.0001 | <0.0001 | <0.0001 | 0.8439 | <0.0001 | >0.9999 |
| ***Crisp2*-WT** | >0.9999 | <0.0001 |  | <0.0001 | >0.9999 | <0.0001 | >0.9999 | 0.1927 | 0.3866 | <0.0001 |  | <0.0001 | 0.5661 | <0.0001 | 0.7749 | <0.0001 | 0.8819 | <0.0001 |  | <0.0001 | >0.9999 | <0.0001 | >0.9999 | <0.0001 |
| ***Crisp2*-KO** | <0.0001 | >0.9999 | <0.0001 |  | <0.0001 | >0.9999 | <0.0001 | 0.0256 | <0.0001 | <0.0001 | <0.0001 |  | <0.0001 | >0.9999 | <0.0001 | 0.1869 | <0.0001 | <0.0001 | <0.0001 |  | <0.0001 | 0.0158 | <0.0001 | <0.0001 |
| ***Crisp4*-WT** | 0.999 | <0.0001 | >0.9999 | <0.0001 |  | <0.0001 | 0.9981 | 0.0942 | >0.9999 | <0.0001 | 0.5661 | <0.0001 |  | <0.0001 | >0.9999 | <0.0001 | 0.8056 | <0.0001 | >0.9999 | <0.0001 |  | <0.0001 | >0.9999 | <0.0001 |
| ***Crisp4*-KO** | <0.0001 | >0.9999 | <0.0001 | >0.9999 | <0.0001 |  | <0.0001 | 0.0256 | <0.0001 | <0.0001 | <0.0001 | >0.9999 | <0.0001 |  | <0.0001 | 0.1869 | <0.0001 | 0.8439 | <0.0001 | 0.0158 | <0.0001 |  | <0.0001 | 0.8941 |
| ***Crisp1/4*-WT** | >0.9999 | <0.0001 | >0.9999 | <0.0001 | 0.9981 | <0.0001 |  | <0.0001 | 0.999 | <0.0001 | 0.7749 | <0.0001 | >0.9999 | <0.0001 |  | <0.0001 | 0.9258 | <0.0001 | >0.9999 | <0.0001 | >0.9999 | <0.0001 |  | <0.0001 |
| ***Crisp1/4*-KO** | 0.3411 | 0.0467 | 0.1927 | 0.0256 | 0.0942 | 0.0256 | <0.0001 |  | <0.0001 | 0.3263 | <0.0001 | 0.1869 | <0.0001 | 0.1869 | <0.0001 |  | <0.0001 | >0.9999 | <0.0001 | <0.0001 | <0.0001 | 0.8941 | <0.0001 |  |
|  | **Mid-piece** | | | | | | | | **Principal piece** | | | | | | | | **End piece** | | | | | | | |

**the shaded cells indicate statistically non-significant interactions* *between wild type combinations.*

**Supplementary Table 5.** Analysis of the total power dissipated per cycle (in ± SD) across *Crisp* wild type and knock out mice.

| **Mouse strain** | **Wild type ± SD** | **Knock-out ± SD** | **P value** |
| --- | --- | --- | --- |
| *Crisp1* | 59 ± 9.5 | 23 ± 9.9 | <0.0001 |
| *Crisp2* | 54 ± 8.7 | 33 ± 5.0 | <0.0001 |
| *Crisp4* | 59 ± 8.2 | 23 ± 4.3 | <0.0001 |
| *Crisp1/4* | 56 ± 1.8 | 26 ± 7.6 | <0.0001 |
| *Please refer to Supplementary Table 5.1 for all the possible interactions between wild type vs. knock out genotype* | | | |

**Supplementary Table 5.1.** Analysis of the total power dissipated per cycle across *Crisp* wild type and knock out mice combinations.

|  | ***Crisp1*-WT** | **C*risp1*-KO** | ***Crisp2*-WT** | ***Crisp2*-KO** | ***Crisp4*-WT** | ***Crisp4*-KO** | ***Crisp1/4*-WT** | ***Crisp1/4*-KO** |
| --- | --- | --- | --- | --- | --- | --- | --- | --- |
| ***Crisp1*-WT** |  | <0.0001 | 0.8672 | <0.0001 | >0.9999 | <0.0001 | >0.9999 | <0.0001 |
| ***Crisp1*-KO** | <0.0001 |  | <0.0001 | 0.0969 | <0.0001 | >0.9999 | <0.0001 | 0.9989 |
| ***Crisp2*-WT** | 0.8672 | <0.0001 |  | <0.0001 | 0.6754 | <0.0001 | 0.9678 | <0.0001 |
| ***Crisp2*-KO** | <0.0001 | 0.0969 | <0.0001 |  | <0.0001 | 0.1022 | <0.0001 | 0.3208 |
| ***Crisp4*-WT** | >0.9999 | <0.0001 | 0.6754 | <0.0001 |  | <0.0001 | 0.997 | <0.0001 |
| ***Crisp4*-KO** | <0.0001 | >0.9999 | <0.0001 | 0.1022 | <0.0001 |  | <0.0001 | 0.9993 |
| ***Crisp1/4*-WT** | >0.9999 | <0.0001 | 0.9678 | <0.0001 | 0.997 | <0.0001 |  | <0.0001 |
| ***Crisp1/4*-KO** | <0.0001 | 0.9989 | <0.0001 | 0.3208 | <0.0001 | 0.9993 | <0.0001 |  |

**the shaded cells indicate statistically non-significant interactions* *between wild type combinations.*

**Supplementary Table 6.** The estimated straight-line velocity (µm/sec) of spermatozoa across *Crisp* wild type and knock out mice.

| **Mouse-line** | **Wild type ± SD** | **Knock-out ± SD** | **P value** |
| --- | --- | --- | --- |
| *Crisp1* | 3.3 ± 0.42 | 2.2 ± 0.35 | < 0.01 |
| *Crisp2* | 3.1 ± 0.27 | 2.7 ± 0.16 | < 0.05 |
| *Crisp4* | 3.3 ± 0.41 | 4.5 ± 0.75 | < 0.01 |
| *Crisp1/4* | 3.5 ± 0.42 | 2.9 ± 0.52 | < 0.01 |

**Supplementary Table 7.1.** Analysis of flagellar amplitude along the different regions of flagella across *Crisp* wild type and knock out mice pre- and post- exposure to recombinant CRISP1 protein.

|  | ***Crisp1*-WT** | ***Crisp1*-WT with rec. CRISP1** | ***Crisp1*-KO** | ***Crisp1*-KO with rec. CRISP1** | ***Crisp1*-WT** | ***Crisp1*-WT with rec. CRISP1** | ***Crisp1*-KO** | ***Crisp1*-KO with rec. CRISP1** | ***Crisp1*-WT** | ***Crisp1*-WT with rec. CRISP1** | ***Crisp1*-KO** | ***Crisp1*-KO with rec. CRISP1** |
| --- | --- | --- | --- | --- | --- | --- | --- | --- | --- | --- | --- | --- |
| ***Crisp1*-WT** |  | 0.9833 | 0.0199 | 0.5144 |  | 0.9815 | 0.1359 | 0.1234 |  | 0.6676 | <0.0001 | 0.1078 |
| ***Crisp1*-WT with rec. CRISP1** | 0.9833 |  | 0.0067 | 0.7409 | 0.9815 |  | 0.0572 | 0.2579 | 0.6676 |  | <0.0001 | 0.6605 |
| ***Crisp1*-KO** | 0.0199 | 0.0067 |  | 0.0002 | 0.1359 | 0.0572 |  | 0.0001 | <0.0001 | <0.0001 |  | <0.0001 |
| ***Crisp1*-KO with rec. CRISP1** | 0.5144 | 0.7409 | 0.0002 |  | 0.1234 | 0.2579 | 0.0001 |  | 0.1078 | 0.6605 | <0.0001 |  |
|  | **Mid-Piece** | | | | **Principle piece** | | | | **End Piece** | | | |

**the shaded cells indicate statistically non-significant interactions.*

**Supplementary Table 7.2.** Analysis of power dissipation (10^-12^ J/s) per beat cycle along the different regions of flagella across *Crisp* wild type and knock out mice pre- and post- exposure to recombinant CRISP1 protein.

|  | ***Crisp1*-WT** | ***Crisp1*-WT with rec. CRISP1** | ***Crisp1*-KO** | ***Crisp1*-KO with rec. CRISP1** | ***Crisp1*-WT** | ***Crisp1*-WT with rec. CRISP1** | ***Crisp1*-KO** | ***Crisp1*-KO with rec. CRISP1** | ***Crisp1*-WT** | ***Crisp1*-WT with rec. CRISP1** | ***Crisp1*-KO** | ***Crisp1*-KO with rec. CRISP1** |
| --- | --- | --- | --- | --- | --- | --- | --- | --- | --- | --- | --- | --- |
| ***Crisp1*-WT** |  | 0.9455 | 0.1965 | 0.7471 |  | 0.505 | <0.0001 | 0.9398 |  | 0.505 | <0.0001 | 0.9398 |
| ***Crisp1*-WT with rec. CRISP1** | 0.9455 |  | 0.0573 | 0.4057 | 0.505 |  | <0.0001 | 0.2044 | 0.505 |  | <0.0001 | 0.4544 |
| ***Crisp1*-KO** | 0.1965 | 0.0573 |  | 0.7589 | <0.0001 | <0.0001 |  | <0.0001 | <0.0001 | <0.0001 |  | 0.0108 |
| ***Crisp1*-KO with rec. CRISP1** | 0.7471 | 0.4057 | 0.7589 |  | 0.9398 | 0.2044 | <0.0001 |  | 0.9398 | 0.4544 | 0.0108 |  |
|  | **Mid-Piece** | | | | **Principle piece** | | | | **End Piece** | | | |

**the shaded cells indicate statistically non-significant interactions.*

**Supplementary Table 8.1.** Analysis of flagellar amplitude along the different regions of flagella across *Crisp* wild type and knock out mice pre- and post- exposure to recombinant CRISP4 protein.

|  | ***Crisp4*-WT** | ***Crisp4*-WT with rec. CRISP4** | ***Crisp4*-KO** | ***Crisp4*-KO with rec. CRISP4** | ***Crisp4*-WT** | ***Crisp4*-WT with rec. CRISP4** | ***Crisp4*-KO** | ***Crisp4*-KO with rec. CRISP4** | ***Crisp4*-WT** | ***Crisp4*-WT with rec. CRISP4** | ***Crisp4*-KO** | ***Crisp4*-KO with rec. CRISP4** |
| --- | --- | --- | --- | --- | --- | --- | --- | --- | --- | --- | --- | --- |
| ***Crisp4*-WT** |  | 0.971 | 0.0284 | 0.7161 |  | 0.705 | 0.0285 | 0.9273 |  | 0.9997 | <0.0001 | 0.0268 |
| ***Crisp4*-WT with rec. CRISP4** | 0.971 |  | 0.12 | 0.9273 | 0.705 |  | 0.0246 | 0.9674 | 0.9997 |  | <0.0001 | 0.0346 |
| ***Crisp4*-KO** | 0.0284 | 0.12 |  | 0.0257 | 0.0285 | 0.0246 |  | 0.0816 | <0.0001 | <0.0001 |  | 0.0002 |
| ***Crisp4*-KO with rec. CRISP4** | 0.7161 | 0.9273 | 0.0257 |  | 0.9273 | 0.9674 | 0.0816 |  | 0.0268 | 0.0346 | 0.0002 |  |
|  | **Mid-Piece** | | | | **Principle piece** | | | | **End Piece** | | | |

**the shaded cells indicate statistically non-significant interactions.*

**Supplementary Table 8.2.** Analysis of power dissipation (10^-12^ J/s) per beat cycle along the different regions of flagella across *Crisp* wild type and knock out mice pre- and post- exposure to recombinant CRISP4 protein.

|  | ***Crisp4*-WT** | ***Crisp4*-WT with rec. CRISP4** | ***Crisp4*-KO** | ***Crisp4*-KO with rec. CRISP4** | ***Crisp4*-WT** | ***Crisp4*-WT with rec. CRISP4** | ***Crisp4*-KO** | ***Crisp4*-KO with rec. CRISP4** | ***Crisp4*-WT** | ***Crisp4*-WT with rec. CRISP4** | ***Crisp4*-KO** | ***Crisp4*-KO with rec. CRISP4** |
| --- | --- | --- | --- | --- | --- | --- | --- | --- | --- | --- | --- | --- |
| ***Crisp4*-WT** |  | 0.5079 | 0.0193 | 0.5079 |  | 0.9383 | <0.0001 | 0.9383 |  | 0.8671 | <0.0001 | 0.0378 |
| ***Crisp4*-WT with rec. CRISP4** | 0.5079 |  | 0.4083 | >0.9999 | 0.9383 |  | <0.0001 | >0.9999 | 0.8671 |  | <0.0001 | 0.2197 |
| ***Crisp4*-KO** | 0.0193 | 0.4083 |  | 0.4083 | <0.0001 | <0.0001 |  | <0.0001 | <0.0001 | <0.0001 |  | <0.0001 |
| ***Crisp4*-KO with rec. CRISP4** | 0.5079 | >0.9999 | 0.4083 |  | 0.9383 | >0.9999 | <0.0001 |  | 0.0378 | 0.2197 | <0.0001 |  |
|  | **Mid-Piece** | | | | **Principle piece** | | | | **End Piece** | | | |

**the shaded cells indicate statistically non-significant interactions.*

**Supplementary Table 9.1.** Analysis of flagellar amplitude along the different regions of flagella across *Crisp* wild type and *Crisp1* knock out mice pre- and post- exposure to recombinant CRISP4 protein and *visa versa.*

|  | **WT** | ***Crisp1*-KO** | ***Crisp1*-KO with rec. CRISP4** | ***Crisp4*-KO** | ***Crisp4*-KO with rec. CRISP4** | **WT** | ***Crisp1*-KO** | ***Crisp1*-KO with rec. CRISP4** | ***Crisp4*-KO** | ***Crisp4*-KO with rec. CRISP4** | **WT** | ***Crisp1*-KO** | ***Crisp1*-KO with rec. CRISP4** | ***Crisp4*-KO** | ***Crisp4*-KO with rec. CRISP4** |
| --- | --- | --- | --- | --- | --- | --- | --- | --- | --- | --- | --- | --- | --- | --- | --- |
| **WT** |  | 0.5951 | 0.3393 | 0.1208 | 0.2463 |  | 0.0009 | 0.0018 | 0.1158 | 0.5682 |  | <0.0001 | <0.0001 | <0.0001 | <0.0001 |
| ***Crisp1*-KO** | 0.5951 |  | 0.996 | 0.9171 | 0.9837 | 0.0009 |  | 0.9998 | 0.6304 | 0.1764 | <0.0001 |  | 0.7324 | 0.4713 | 0.994 |
| ***Crisp1*-KO with rec. CRISP4** | 0.3393 | 0.996 |  | 0.9898 | 0.9998 | 0.0018 | 0.9998 |  | 0.7324 | 0.2438 | <0.0001 | 0.7324 |  | 0.994 | 0.9238 |
| ***Crisp4*-KO** | 0.1208 | 0.9171 | 0.9898 |  | 0.998 | 0.1158 | 0.6304 | 0.7324 |  | 0.9238 | <0.0001 | 0.4713 | 0.994 |  | 0.7324 |
| ***Crisp4*-KO with rec. CRISP4** | 0.2463 | 0.9837 | 0.9998 | 0.998 |  | 0.5682 | 0.1764 | 0.2438 | 0.9238 |  | <0.0001 | 0.994 | 0.9238 | 0.7324 |  |
|  | **Mid-piece** | | | | | **Principle piece** | | | | | **End Piece** | | | | |

**the shaded cells indicate statistically non-significant interactions.*

**Supplementary Table 9.2.** Analysis of power dissipation (10^-12^ J/s) per beat cycle along the different regions of flagella across *Crisp* wild type and *Crisp1* knock out mice pre- and post- exposure to recombinant CRISP4 protein *visa versa*.

|  | **WT** | ***Crisp1*-KO** | ***Crisp1*-KO with rec. CRISP4** | ***Crisp4*-KO** | ***Crisp4*-KO with rec. CRISP4** | **WT** | ***Crisp1*-KO** | ***Crisp1*-KO with rec. CRISP4** | ***Crisp4*-KO** | ***Crisp4*-KO with rec. CRISP4** | **WT** | ***Crisp1*-KO** | ***Crisp1*-KO with rec. CRISP4** | ***Crisp4*-KO** | ***Crisp4*-KO with rec. CRISP4** |
| --- | --- | --- | --- | --- | --- | --- | --- | --- | --- | --- | --- | --- | --- | --- | --- |
| **WT** |  | 0.2103 | 0.1143 | 0.0599 | 0.0836 |  | <0.0001 | <0.0001 | <0.0001 | <0.0001 |  | <0.0001 | <0.0001 | <0.0001 | 0.001 |
| ***Crisp1*-KO** | 0.2103 |  | 0.9991 | 0.9881 | 0.9958 | <0.0001 |  | 0.9841 | 0.8797 | >0.9999 | <0.0001 |  | 0.9983 | 0.9958 | 0.8952 |
| ***Crisp1*-KO with rec. CRISP4** | 0.1143 | 0.9991 |  | 0.9958 | 0.9993 | <0.0001 | 0.9841 |  | 0.9939 | 0.9949 | <0.0001 | 0.9983 |  | 0.963 | 0.7521 |
| ***Crisp4*-KO** | 0.0599 | 0.9881 | 0.9958 |  | >0.9999 | <0.0001 | 0.8797 | 0.9939 |  | 0.9287 | <0.0001 | 0.9958 | 0.963 |  | 0.9841 |
| ***Crisp4*-KO with rec. CRISP4** | 0.0836 | 0.9958 | 0.9993 | >0.9999 |  | <0.0001 | >0.9999 | 0.9949 | 0.9287 |  | 0.001 | 0.8952 | 0.7521 | 0.9841 |  |
|  | **Mid-piece** | | | | | **Principle piece** | | | | | **End Piece** | | | | |

**the shaded cells indicate statistically non-significant interactions.*

**Supplementary Table 10.1.** Analysis of flagellar amplitude along the different regions of flagella across *Crisp1/4* double knock out mice pre- and post- exposure to individual recombinant CRISP1. CRISP4 and combined CRISP1 and 4 proteins.

|  | **WT** | ***Crisp1/4*-KO** | ***Crisp1/4*-KO with rec. CRISP1** | ***Crisp1/4*-KO with rec. CRISP4** | ***Crisp1/4*-KO with rec. CRISP1 and CRISP4** | **WT** | ***Crisp1/4*-KO** | ***Crisp1/4*-KO with rec. CRISP1** | ***Crisp1/4*-KO with rec. CRISP4** | ***Crisp1/4*-KO with rec. CRISP1 and CRISP4** | **WT** | ***Crisp1/4*-KO** | ***Crisp1/4*-KO with rec. CRISP1** | ***Crisp1/4*-KO with rec. CRISP4** | ***Crisp1/4*-KO with rec. CRISP1 and CRISP4** |
| --- | --- | --- | --- | --- | --- | --- | --- | --- | --- | --- | --- | --- | --- | --- | --- |
| **WT** |  | 0.9996 | 0.9897 | 0.9687 | 0.971 |  | 0.9984 | 0.9932 | >0.9999 | 0.9996 |  | 0.7934 | >0.9999 | 0.4889 | 0.7934 |
| ***Crisp1/4*-KO** | 0.9996 |  | 0.9993 | 0.9952 | 0.9958 | 0.9984 |  | >0.9999 | 0.9987 | >0.9999 | 0.7934 |  | 0.8178 | 0.9922 | >0.9999 |
| ***Crisp1/4*-KO with rec. CRISP1** | 0.9897 | 0.9993 |  | 0.9999 | >0.9999 | 0.9932 | >0.9999 |  | 0.9946 | 0.9996 | >0.9999 | 0.8178 |  | 0.5525 | 0.8178 |
| ***Crisp1/4*-KO with rec. CRISP4** | 0.9687 | 0.9952 | 0.9999 |  | >0.9999 | >0.9999 | 0.9987 | 0.9946 |  | 0.9996 | 0.4889 | 0.9922 | 0.5525 |  | 0.9922 |
| ***Crisp1/4*-KO with rec. CRISP1 and CRISP4** | 0.971 | 0.9958 | >0.9999 | >0.9999 |  | 0.9996 | >0.9999 | 0.9996 | 0.9996 |  | 0.7934 | >0.9999 | 0.8178 | 0.9922 |  |
|  | **Mid-piece** | | | | | **Principle piece** | | | | | **End Piece** | | | | |

**the shaded cells indicate statistically non-significant interactions* *between combinations.*

**Supplementary Table 10.2.** Analysis of power dissipation (10^-12^ J/s) per beat cycle along the different regions of flagella across *Crisp1/4* double knock out mice pre- and post- exposure to individual recombinant CRISP1. CRISP4 and combined CRISP1 and 4 proteins.

|  | **WT** | ***Crisp1/4*-KO** | ***Crisp1/4*-KO with rec. CRISP1** | ***Crisp1/4*-KO with rec. CRISP4** | ***Crisp1/4*-KO with rec. CRISP1 and CRISP4** | **WT** | ***Crisp1/4*-KO** | ***Crisp1/4*-KO with rec. CRISP1** | ***Crisp1/4*-KO with rec. CRISP4** | ***Crisp1/4*-KO with rec. CRISP1 and CRISP4** | **WT** | ***Crisp1/4*-KO** | ***Crisp1/4*-KO with rec. CRISP1** | ***Crisp1/4*-KO with rec. CRISP4** | ***Crisp1/4*-KO with rec. CRISP1 and CRISP4** |
| --- | --- | --- | --- | --- | --- | --- | --- | --- | --- | --- | --- | --- | --- | --- | --- |
| **WT** |  | 0.6262 | 0.872 | 0.9434 | 0.9797 |  | <0.0001 | <0.0001 | 0.0107 | 0.0681 |  | <0.0001 | <0.0001 | <0.0001 | 0.0004 |
| ***Crisp1/4*-KO** | 0.6262 |  | 0.9948 | 0.9778 | 0.9745 | <0.0001 |  | 0.9989 | 0.467 | 0.4459 | <0.0001 |  | 0.9459 | 0.8336 | 0.4984 |
| ***Crisp1/4*-KO with rec. CRISP1** | 0.872 | 0.9948 |  | 0.9997 | 0.9991 | <0.0001 | 0.9989 |  | 0.311 | 0.3081 | <0.0001 | 0.9459 |  | 0.9982 | 0.8828 |
| ***Crisp1/4*-KO with rec. CRISP4** | 0.9434 | 0.9778 | 0.9997 |  | >0.9999 | 0.0107 | 0.467 | 0.311 |  | 0.9996 | <0.0001 | 0.8336 | 0.9982 |  | 0.9636 |
| ***Crisp1/4*-KO with rec. CRISP1 and CRISP4** | 0.9797 | 0.9745 | 0.9991 | >0.9999 |  | 0.0681 | 0.4459 | 0.3081 | 0.9996 |  | 0.0004 | 0.4984 | 0.8828 | 0.9636 |  |
|  | **Mid-piece** | | | | | **Principle piece** | | | | | **End Piece** | | | | |

**the shaded cells indicate statistically non-significant interactions* *between combinations.*
